# Insights into SGLT2 inhibitor treatment of diabetic cardiomyopathy: focus on the mechanisms

**DOI:** 10.1186/s12933-023-01816-5

**Published:** 2023-04-13

**Authors:** Keming Huang, Xianling Luo, Bin Liao, Guang Li, Jian Feng

**Affiliations:** 1 Department of Cardiology, The Affiliated Hospital of Southwest Medical University, Key Laboratory of Medical Electrophysiology, Ministry of Education and Medical Electrophysiological Key Laboratory of Sichuan Province, Institute of Cardiovascular Research, Southwest Medical University, Luzhou, Sichuan China; 2 Department of Cardiovascular Surgery, Metabolic Vascular Diseases Key Laboratory of Sichuan Province, The Affiliated Hospital of Southwest Medical University, Luzhou, Sichuan China

**Keywords:** Sodium-glucose cotransporter 2 inhibitor, Diabetic cardiomyopathy, Mechanisms

## Abstract

Among the complications of diabetes, cardiovascular events and cardiac insufficiency are considered two of the most important causes of death. Experimental and clinical evidence supports the effectiveness of SGLT2i for improving cardiac dysfunction. SGLT2i treatment benefits metabolism, microcirculation, mitochondrial function, fibrosis, oxidative stress, endoplasmic reticulum stress, programmed cell death, autophagy, and the intestinal flora, which are involved in diabetic cardiomyopathy. This review summarizes the current knowledge of the mechanisms of SGLT2i for the treatment of diabetic cardiomyopathy.

**Graphical Abstract:**

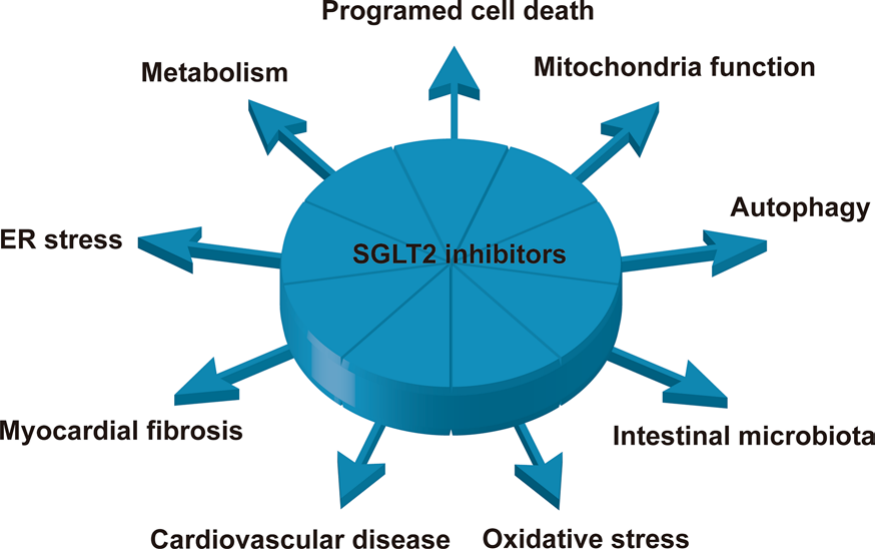

## Introduction

Diabetes may have been recognized more than 3000 years ago [1], and the incidence and prevalence of this ancient disease have increased with improvements in living and health standards. Currently, 1 in 11 people has diabetes, which is 4 times more frequent than 30 years ago [2]. In addition to eye, kidney, and foot disease, chronic hyperglycaemia can damage the myocardium, which manifests as diabetic cardiomyopathy. The pathology of diabetic cardiomyopathy includes abnormal carbohydrate and fatty acid metabolism, microvascular disease, endothelial dysfunction, mitochondrial dysfunction, myocardial diastolic dysfunction and fibrosis, excessive cardiomyocyte programmed cell death, increased oxidative stress, endoplasmic reticulum (ER) stress, and glycotoxicity [3–5]. Adverse responses to abnormal blood glucose lead to structural remodelling and impaired function in the heart. Diabetic cardiomyopathy is known to significantly increase the risk of heart failure and can lead to a preserved or reduced ejection fraction [6].

Sodium-glucose cotransporter 2 inhibitors (SGLT2is) have long been used as drugs to treat diabetes and have recently been shown to improve cardiac function in diabetes. In a randomized controlled trial, dapagliflozin reduced heart failure by 27% in T2DM (type 2 diabetes mellitus) patients with cardiovascular disease. The effectiveness was 35% for empagliflozin and 33% for canagliflozin [7]. This review focuses on the benefits of SGLT2i for cardiomyopathy in diabetes, including the mechanism (Table 1). Future prospects are also discussed.

**Table 1:** Therapeutic targets of SGLT2i in basic science trials

Drug	Target	Organ or tissue	Model	Effect
Empagliflozin	GLUT1	Heart	Isolated failing human and murine cardiomyocytes	Improve glucose metabolism [1]
Empagliflozin	HMGCS2	Liver, kidney and jejunum	Normal and db/db mice	Increase serum ketone bodies [2]
Empagliflozin	CPT1b	Heart	Otsuka long-evans tokushima fatty rats	Reduce fatty acid utilization [3]
Empagliflozin	PPARγ/CD36	Heart	Zucker diabetic fatty rats	Reduce the accumulation of fatty acids [4]
Canagliflozin	PPAR-α	Adipose tissue	Obese mice due to a high-fat diet	Decrease plasma TG and TC [5]
Dapagliflozin	Drp1	Heart	High-fat diet-induced insulin-resistant obesity rats	Inhibit mitochondrial fission [6]
Dapagliflozin, Empagliflozin	MFN1/MFN2 and OPA1	Heart	Metabolic syndrome rats; high-fat diet/STZ-induced diabetic rats	Inhibit mitochondrial fission [7, 8]
Empagliflozin	PGC-1a, NRF-1 and mtTFA	Heart	High-fat diet/STZ-induced diabetic rats	Promote mitochondrial biogenesis [8]
Empagliflozin, Dapagliflozin, Canagliflozin	ETC complex I and II	Human RPTEC/TERT1 cells	Normal	Improve the activity of ETC complex I and II (Canagliflozin reduce the activity of ETC complex I) [9]
Dapagliflozin	O-GlcNAc transferase	Kidney	STZ-induced diabetic rats	May improve the activity of ETC complexes [10]
Empagliflozin	Phenotype polarization of macrophages	The aorta	Mouse model of atherosclerosis with diabetes	Reduce atherosclerosis [11, 12]
Empagliflozin	AT1R and ACE	Coronary artery endothelium	High glucose-treated porcine coronary artery	Delay endothelial cell senescence [13]
Luseogliflozin	GLUT9 isoform 2	*Xenopus laevis* oocytes	Cells were injected with 0.1–25 ng of cRNA of GLUT9 isoform 2	Reduce uric acid [14]
Empagliflozin	COX-2	The aorta	STZ-induced diabetes rats	Improve vascular dysfunction [15]
Dapagliflozin	PKG/Kv channels	The rabbit aorta	Normal	Improve vascular dysfunction [16]
Dapagliflozin; empagliflozin	TNF-α/ROS/NO	Human coronary arterial endothelial cells	TNF-α stimulation	Improve vascular dysfunction [17]
empagliflozin	L-arginine/NO	Coronary arteries	ob/ob^−/−^ mice	Improve vascular dysfunction [18]
Empagliflozin; canagliflozin	NHE1	Coronary arteries	Normal mice	Improve vascular dysfunction [19]
Empagliflozin	AMP/ATP/AMPK/Drp1	Heart	STZ-induced diabetic mice	Increase the number of CD31 + microvessels [20]
Empagliflozin	AGEs/RAGE/PKC-ζ/MAPK	Kidney	STZ-induced diabetic rats	Inhibit fibrosis [21]
Empagliflozin	TGF-β/SMAD	Heart	Genetic type 2 diabetes mouse model	Inhibit fibrosis [22]
Dapagliflozin	STAT3	Heart	Myocardial infarction in rats	Inhibit fibrosis [23]
Empagliflozin	NO/sGC-cGMP/PKG pathway [24, 25]	Heart	Human and murine HFpEF myocardium	Reduce cardiomyocyte stiffness [26]
Empagliflozin	Nrf2/ARE pathway	Heart	Genetic type 2 diabetes mouse model	Inhibit oxidative stress [22]
Canagliflozin	AMPK/Akt/eNOS	Heart	ISO-induced oxidative stress in rats	Inhibit oxidative stress [27]
Canagliflozin	iNOS, NOX4	Heart	ISO-induced oxidative stress in rats	Inhibit oxidative stress [27]
Empagliflozin	Sirt1/Nrf2; Sirt1/Foxo1 [28, 29]	Liver and kidney	Otsuka long-evans tokushima fatty rats, rats with induced insulin resistance	Inhibit oxidative stress [30, 31]
Dapagliflozin, Empagliflozin	ERK/Bax; STAT3/Bcl-2; AMPK/TNF-α; Caspase -3	Heart	LPS-induced inflammation in mouse atrial myocytes; cardiorenal syndrome in rats; rats with cardiac I/R injury	Inhibit apoptosis [12, 32, 33]
Dapagliflozin	AMPK/NLRP3/ASC/caspase-1 pathway	Heart	BTBR ob/ob mice	Inhibit pyroptosis [34]
Empagliflozin	CD36/AMPK/Ulk1/Beclin1	Heart	ZDF rats	Promote autophagy [4, 35]
Empagliflozin	NHE1 and NHE1-related genes	Heart	db/db mice with myocardial infarction	Inhibit autophagy [36]
Empagliflozin	Beclin1	Heart	Myocardial infarction with acute hyperglycaemia in mice	Inhibit autophagy [37]
Dapagliflozin	SIRT1/PERK/eIF2α/ATF4/CHOP	Heart	Heart pressure-overload in mice; myocardial I/R injury in mice	Improve ER stress [38, 39]
Dapagliflozin	Abundance of *Akkermansia muciniphila*	Gut	Diabetic mice homozygous for a point mutation in the leptin receptor gene	Improve glucose tolerance and atherosclerosis [40]
Luseogliflozin, empagliflozin	The abundance of SCFA-producing bacteria	Gut	db/db mice	Improve glucose tolerance and atherosclerosis [41, 42]

## SGLT2i and improved heart function

The first SGLT inhibitor was dihydrochalcone phlorizin, which is a nonselective SGLT inhibitor extracted from apple tree roots [8]. Dihydrochalcone phlorizin contains a glucose moiety and an aglycone in which two aromatic carbocycles are joined by an alkyl spacer. Later, the aromatic O-glycoside sergliflozin and the aromatic C-glycoside dapagliflozin officially opened the era of selective SGLT inhibitors [9]. The available SGLT2i are functionally similar but differ in selectivity, efficacy, and indication. For example, empagliflozin, dapagliflozin, and canagliflozin are 2600, 1200, and 150 times more selective for SGLT2 than for SGLT1 [10]. Ipragliflozin and dapagliflozin were approved to treat T1DM (type 1 diabetes mellitus) and T2DM, but most other SGLT2i were approved to treat only T2DM [10]. There are also some obvious differences among them; for example, compared with dapagliflozin and empagliflozin, canagliflozin has a stronger inhibitory effect on angiogenesis [11, 12].

The ways in which SGLT2 functions in the heart are not fully understood. SGLT1 is expressed by cardiomyocytes and may be a target of SGLT2i that improves heart failure [13]. Inhibiting SGLT1 with canagliflozin was shown to reduce nicotinamide adenine dinucleotide phosphate (NADPH) oxidase activity in cardiomyocytes, thereby inhibiting the production of superoxide and decreasing inflammation [13]. SGLT2i may be useful as a first-line treatment for heart failure that is not only mediated by their target receptors. For example, dapagliflozin and canagliflozin were reported to directly inhibit Na^+^/H^+^ exchanger-1 (NHE1) and abrogate the increase in cytosolic Na^+^ in cardiomyocytes. Direct activation of AMP-activated protein kinase (AMPK) by dapagliflozin could reduce lipopolysaccharide (LPS)-induced myocardial fibrosis. That kind of intermolecular interaction is not dependent on SGLT2i-mediated inhibition of the sodium-glucose cotransporter [14]. Other studies have shown that the cardiac sodium channel Nav1.5 was a target of SGLT2i and that inhibiting sodium channels ameliorated dysfunctional sodium and calcium homeostasis, improved calcium overload, and decreased the incidence of malignant arrhythmias [15]. Ongoing study of SGLT2i is expected to reveal additional mechanisms of action.

## SGLT2i and the regulation of heart metabolism

Disorders of glucose and fatty acid metabolism are prominent features of diabetic cardiomyopathy. Under normal conditions, fatty acids are the first-choice energy source and account for 70–90% of the ATP produced by cardiomyocytes. Although each molecule of fatty acid produces more ATP than glucose, complete oxidation requires more oxygen. When the same amount of oxygen is consumed, fatty acids produce less ATP than glucose (2.33 vs. 2.58) [16]. However, compared with the absence of diabetes, oxidative metabolism in cardiomyocytes under diabetic conditions consumes a higher percentage of fatty acids and a lower percentage of glucose [17], and there is an increase in the use of ketone bodies [18]. The presence of diabetes increases oxygen consumption and decreases efficiency in the heart [17].

### SGLT2i and myocardial glucose utilization

Evidence of the regulation of glucose uptake and utilization by SGLT2i may provide ideas for further research. The glucose transporter (GLUT) isoforms GLUT4 and GLUT1 are predominant in the heart [19]. The mechanism of action is not known, but data show that SGLT2i benefit myocardial energy metabolism. Empagliflozin has been shown to increase glucose uptake in the human and murine myocardium that was associated with increased GLUT1 expression [20]. Empagliflozin was also reported to increase glycolysis and glucose oxidation rates in the myocardium of db/db mice [21].

### SGLT2i and myocardial fat metabolism

The dominant carnitine O-palmitoyltransferase (CPT) isoform in the heart is CPT1b. It is located on the outer mitochondrial membrane and catalyses carnitine conjugation of long-chain fatty acids, which facilitates mitochondrial transport and β-oxidation in cardiomyocytes [22]. In a rat diabetes model, CPT1b expression in myocardial tissues was significantly increased compared with that in nondiabetic controls. As previously stated, increased fatty acid utilization increases the oxygen requirements of cardiomyocytes. However, the mRNA and protein expression of CPT1b was reduced by subcutaneous injection of empagliflozin [23], which indicates that SGLT2i improved cardiomyocyte energy metabolism by reducing fatty acid utilization.

Peroxisome proliferator-activated receptors (PPARs) are a superfamily of nuclear receptor proteins that act as transcription factors and have at least three isoforms: α, δ and γ. Empagliflozin inhibits the mRNA and protein expression of PPAR-γ in the kidney [24]. Most likely because of the same effect, it decreases the expression of CD36, which is the downstream molecule of PPAR-γ, in cardiac tissue [25] and reduces the uptake and accumulation of fatty acids [26]. When activated, PPAR-α promotes the use of fatty acids and reduces the use of glucose [27]. SGLT2i do not directly decrease PPAR-α mRNA expression in the myocardium [23] but promote PPAR-α mRNA and protein expression in adipose tissue and decrease the concentrations of triglycerides (TG) and total cholesterol (TC) in plasma [28]. Adiponectin is a peptide that is secreted by adipocytes, and its effects on lipid metabolism are reflected by a negative correlation with serum TG and association with increased utilization of glucose and fatty acids by muscle tissue [29]. A systematic review and meta-analysis of randomized controlled trials showed that SGLT2i increased adiponectin levels in T2DM [30]. It has been proven that abnormal TC and TG levels are closely related to diabetes [31]. The control of lipid metabolism not only helps to prevent cardiovascular events but also improves left ventricle systolic dysfunction [32, 33].

### SGLT2i and myocardial ketone body metabolism

Fatty acids and glucose are the major fuels in the normal heart. Ketone bodies are a minor energy source. In diabetes patients, insulin resistance decreases glucose transport and availability as an energy source. As a result, cardiomyocytes increase their utilization of the ketone body beta-hydroxybutyrate (β-OHB) [18]. SGLT2i have been shown to increase myocardial utilization of ketone bodies to increase ATP production. Verma et al. [21] showed that overall cardiac ATP production was 36% lower in db/db mice than in C57BL/6 J mice and that empagliflozin increased cardiac ATP production by 31% compared with that in untreated db/db mice. Strangely, empagliflozin did not directly improve the efficiency of myocardial ketone body utilization. However, it has been reported that empagliflozin can increase serum ketone concentrations by activating the expression of the ketogenic enzyme HMGCS2 in other tissues [34]. Moreover, the frugal fuel hypothesis suggests that an increase in ketone body concentration is responsible for the increased efficiency of cardiac mitochondrial oxidation in response to empagliflozin [35, 36]. In a subsequent experiment on the perfusion of isolated mouse hearts, researchers found that the addition of β-OHB to the system produced effects similar to those observed in response to empagliflozin. Therefore, we can conclude that empagliflozin increases blood ketone concentrations, thereby increasing overall ketone utilization. This result was subsequently confirmed by a similar study [37].

## SGLT2i and myocardial mitochondria

Aerobic respiration that occurs in the mitochondria of cardiomyocytes provides energy for cardiac contraction. Mitochondrial dysfunction occurs in diabetic cardiomyopathy as a consequence of high glucose, insulin resistance, and obesity. The consequences of these pathological changes include but are not limited to increased mitochondrial fission, autophagy, reduced oxidative phosphorylation and impaired ATP production, the accumulation of metabolic intermediates and reactive oxygen species (ROS), oxidative stress, apoptosis, and impaired mechanical function in the heart [38–40].

**Fig. 1:**
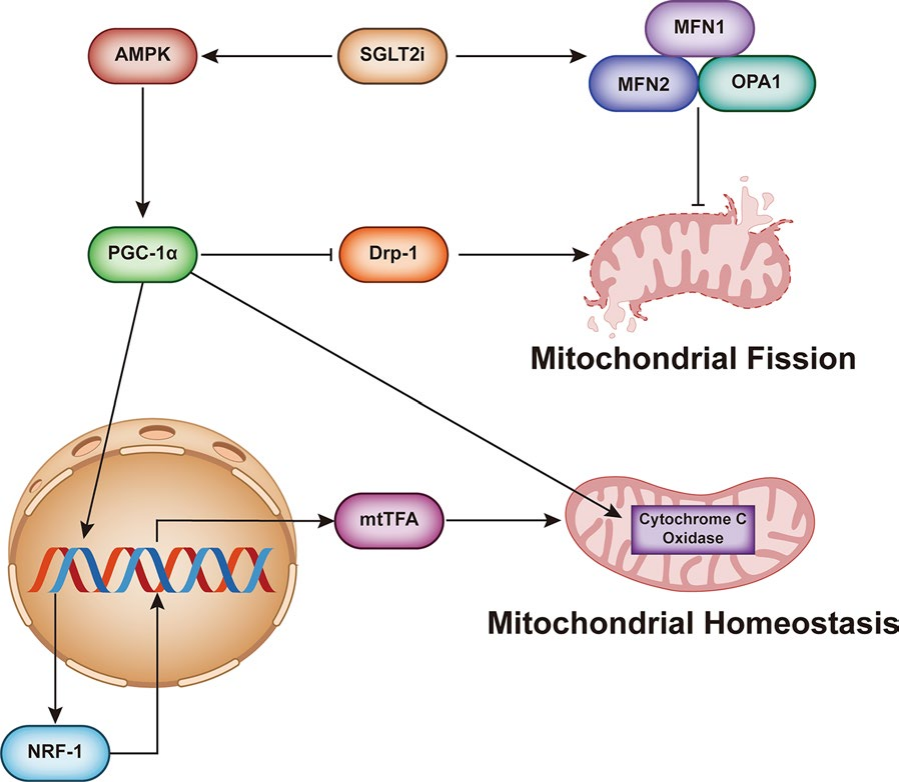
SGLT2 inhibitors improve mitochondrial function. SGLT2 inhibitors activate the AMPK/PGC-1α signalling pathway, thereby increasing PGC-1α-regulated NRF-1 expression, which increases mtTFA expression and further promotes mitochondrial biogenesis. PGC-1α can also inhibit Drp1-induced mitochondrial fission and activate key enzymes, including cytochrome c oxidase, in mitochondria, which can increase the efficiency of electron transfer. Activation of AMPK, on the other hand, leads to the recovery of abnormal MFN1, MFN2, and OPA1, reducing mitochondrial fission

Mitochondria frequently divide and fuse with each other [41]. When cells are subjected to mild stress, mitochondria form an extensive interconnected network. When cells are severely stressed, mitochondria undergo fission and are fragmented [42]. In response to hyperglycaemia, cardiomyocyte mitochondria undergo dynamin-related protein 1 (Drp1)-mediated fission, which, if excessive, can result in fragmentation, ROS production, increased oxidative stress and even cell death [43]. Tanajak et al. [44] studied the inhibitory effect of SGLT2i on excessive mitochondrial fission after cardiac ischaemia/reperfusion (I/R) injury in the hearts of obese rats with high-fat diet-induced insulin resistance. Dapagliflozin reversed the impairment in mitochondrial morphology and increased ROS levels. The total myocardial Drp1 protein level did not change significantly, but the myocardial mitochondrial Drp1 level decreased significantly [44]. The myocardial phosphorylation site Drp1 Ser-637 was activated by dapagliflozin, which may have accounted for the reduction in the mitochondrial translocation of Drp1 [45]. These data are consistent with those of Zhou et al. [46], who reported that the upstream signalling molecule of Drp1 was AMPK. Mitochondrial fusion and fission are also associated with mitofusins (MFNs) and optic atrophy 1 (OPA1). MFN1 and MFN2 mediate fusion of the mitochondrial outer membrane, OPA1 mediates inner membrane fusion [47], and the absence of any one of the three causes cells to produce different types of fragmented mitochondria [48, 49]. Therefore, MFNs and OPA1 are targets of SGLT2i therapy. Durak et al. [50] reported that dapagliflozin normalized the increase in MFN1, decrease in MFN2, and increase in OPA1 expression in the myocardium of rats with sucrose-induced metabolic syndrome. These results have also been reported for empagliflozin [51] (Fig. 1).

Mitochondrial biogenesis and the growth and division of preexisting mitochondria are regulated by AMPK, peroxisome-proliferator-activated receptor γ coactivator-1α (PGC-1α), and nuclear respiratory factors (NRF)-1 and -2 [52]. AMPK activation was shown to increase the transcription of the PGC-1α gene in rat cell nuclei, which then induced the expression of the transcription factor NRF-1 [53]. Mitochondrial transcription factor A (mtTFA) is a mitochondrial promoter that stimulates transcription. The proximal promoter of the human mtTFA gene is dependent on the activity of the recognition sites of the nuclear respiratory factors NRF-1 and NRF-2 [54]. Activation of PGC-1α, NRF-1, and mtTFA increases the transcription and replication of mitochondrial DNA. PGC-1α can also activate the expression of key enzymes in mitochondria, including cytochrome c oxidase [55]. This will allow the mitochondrial electron transport chain (ETC) to increase the efficiency of electronic transfer. These regulators of mitochondrial biogenesis are inhibited to varying degrees in diabetes. However, empagliflozin reversed the downregulation of PGC-1α, NRF-1, and mtTFA in a streptozotocin (STZ)-induced rat model of T2DM [51] (Fig. 1).

The transfer of electrons in the ETC generates mitochondrial membrane potential. The reduced activity of ETC complexes in the hearts of diabetes patients is accompanied by a reduction in the mitochondrial respiration rate [56]. The decreases in mitochondrial state 3 respiration and mitochondrial membrane potential in rats with STZ-induced diabetes were reversed by high-dose empagliflozin [51]. Secker et al. [57] found that canagliflozin inhibited the activity of mitochondrial respiratory complex I and promoted complex II activity, and empagliflozin and dapagliflozin increased the activity of complex I. Improvements in mitochondrial function by SGLT2i may be related to a decrease in O-GlcNAcylation. The increase in O-GlcNAcylation secondary to hyperglycaemia leads to a decrease in the activity of ETC complexes I, III, and IV. This change is mainly due to the O-GlcNAcylated subunits that make up the respiratory chain complexes [58]. Dapagliflozin and other SGLT2i may improve the function of the mitochondrial respiratory chain by directly reducing O-GlcNAc transferase activity and O-GlcNAcylation [59].

## SGLT2i improve cardiovascular disease and microcirculation

Diabetes is known to increase the risk of cardiovascular events and cause myocardial microvascular complications [60, 61]. Damage to vascular endothelial cells caused by oxidative stress is followed by a series of pathological changes that include vascular inflammation, vasoconstriction, thrombosis and atherosclerosis [62]. Diabetes and glycotoxicity promote coronary atherosclerosis by exacerbating endothelial dysfunction and increasing oxidative stress, blood lipids, and autonomic dysfunction [63].

### SGLT2i attenuate vascular inflammation

Vascular inflammation is mediated in part by mitochondrial fission, which is a complex mechanism involving tumour necrosis factor-α (TNF-α), Drp-1, NF-κB, and vascular cell adhesion molecule-1 (VCAM-1) [64]. Primary cultures of rat aortic endothelial cells transduced with an adenovirus encoding a dominant-negative Drp1K38A mutant showed significant inhibition of TNF-α-induced NF-κB-driven promoter activity and VCAM-1 induction. These factors are responsible for chronic persistent inflammation and atherosclerosis [65]. SGLT2i were reported to improve endothelial function in mice with STZ-induced diabetes by inhibiting abnormal mitochondrial fission and endothelial inflammation [46]. The anti-inflammatory effect of empagliflozin on arterial endothelial cells has been linked to the activation of AMPK and inhibition of Drp1 by the phosphorylation of Ser-637 [66].

Macrophage infiltration and polarization towards the M1 phenotype are key events in the development of atherosclerosis [67]. Macrophages can polarize to two phenotypes: M1 and M2. M1 macrophages exacerbate the inflammatory response, and M2 macrophages are involved in the resolution of inflammation [68]. In a mouse model of diabetes with atherosclerosis and hypercholesterolemia, empagliflozin decreased the proliferation of plaque-resident macrophages and reduced the size of atherosclerotic plaques [69]. The mechanism involved reducing M1 phenotype polarization and increasing M2 polarization in response to SGLT2i [70].

SGLT2i have also been reported to delay endothelial cell senescence, and the effect may depend on angiotensin converting enzyme (ACE) activity and angiotensin type 1 receptor (AT1R). Angiotensin II induces endothelial senescence [71], and Khemais-Benkhiat et al.[72] reported that hyperglycaemia increased the protein expression of ACE and AT1R and increased β-galactosidase, a biomarker of cellular senescence[73], in porcine coronary endothelial cells. Empagliflozin reversed these changes in the presence of hyperglycaemia but did not affect ACE or AT1R in control cells in the absence of hyperglycaemia [72].

**Fig. 2:**
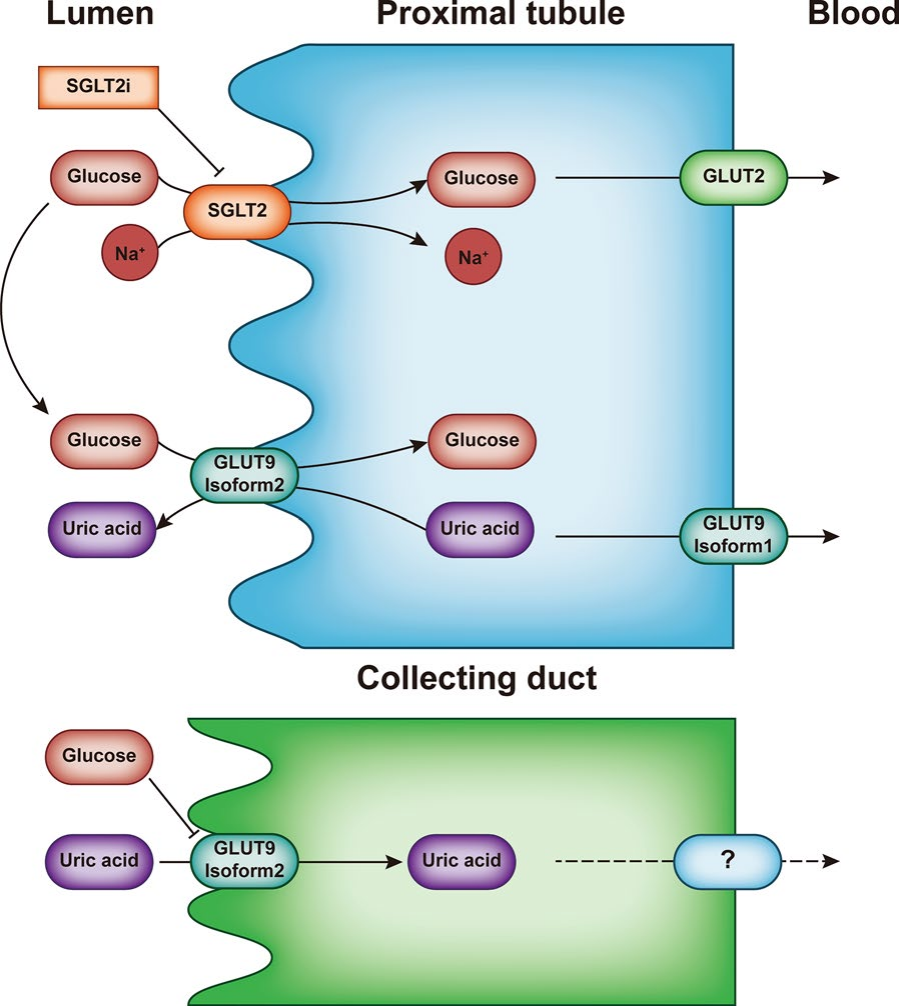
SGLT2i can improve vasodilatory inflammation by decreasing uric acid levels. In proximal renal tubules, SGLT2i acts on SGLT2, decreasing the reabsorption of Na ions and glucose and increasing glucose concentrations in the lumen, which is exchanged with uric acid by GLUT9 isoform 2. This leads to increased uric acid exclusion. In the collecting duct, high levels of glucose inhibit this exchange and reduce the absorption of uric acid, thereby draining it. Lowering the concentration of uric acid in the blood helps reduce inflammation in the blood vessels

Hyperuricaemia is another independent risk factor for diabetes. Uric acid levels increase in the early stages of impaired glucose metabolism, and hyperuricaemia is associated with micro- and macrovascular complications of diabetes [74, 75]. Uric acid concentrations higher than physiological levels inhibit NO synthesis, reduce NO activity and induce NF-κB, leading to the induction of monocyte chemoattractant protein 1 and cyclooxygenase 2 (COX-2), which mediate inflammation and atherosclerosis [76]. Chino et al. [77] found that the SGLT2i luseogliflozin increased uric acid excretion and that GLUT9 isoform 2 was involved. SGLT2i increased the concentration of glucose in the proximal tubule, which caused GLUT9 isoform 2 and other transporters to reabsorb more glucose and excrete more uric acid. In the collecting ducts, a high concentration of glucose prevents GLUT9 isoform 2 from reabsorbing uric acid (Fig. 2).

### SGLT2i regulates diastolic and systolic flow in blood vessels

**Fig. 3:**
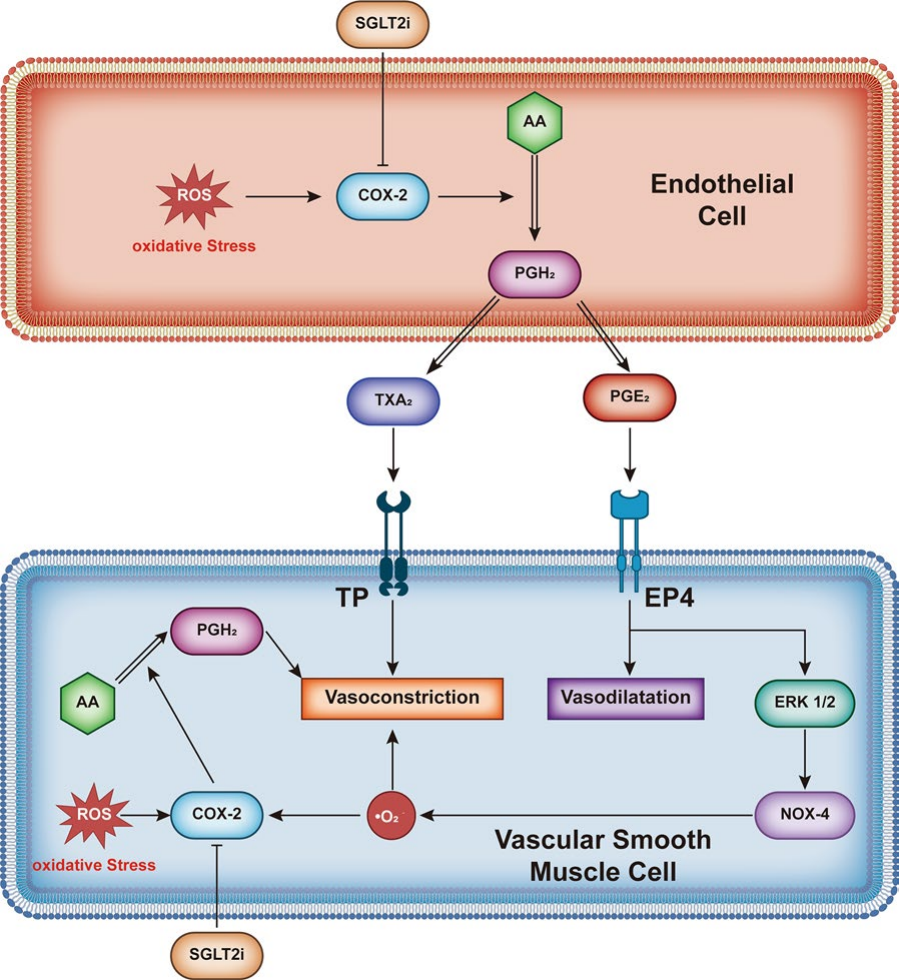
SGLT2i can improve vasodilatory functions through COX-2. In endothelial cells, SGLT2i inhibit the production of ROS-induced PGH2 by inhibiting COX-2 and reduce the production of PGE2 and TXA2 downstream of PGH2. In vascular smooth muscle, although PGE2 activates EP4 receptors to dilate blood vessels, TAX2 activates TP receptors to cause vasoconstriction. In addition, downstream EP4 can cascade into ERK1/2/NOX4 to produce ROS, which contributes to vasoconstriction and the activation of COX-2. In vascular smooth muscle, COX-2 activates and catalyses AA to produce PGH2, which constricts blood vessels

Studies have shown that flow-mediated dilation (FMD) is reduced in young T2DM patients relative to healthy individuals [78], and in coronary arteries, the maximal pharmacologic flow reserve is significantly lower in diabetes patients than in healthy individuals [79]. Dapagliflozin has been shown to improve FMD in patients with T2DM [80], which may depend on the inhibition of COX-2. The increase in ROS production may involve COX-2/prostaglandin E2 (PGE2)/E-type prostaglandin receptor 4 (EP4)/extracellular signal-regulated kinase 1/2 (ERK1/2)/NADPH oxidase isoform 4 (Nox4) signalling [81]. Increased ROS resulting in vasoconstriction involves the initiation of calcium flux and stimulating pathways leading to the sensitization of contractile elements to calcium [82]. Therefore, this kind of vasoconstriction can be inhibited by selective COX-2 inhibitors. SGLT2i inhibit COX-2 mRNA expression and vasoconstriction [83]. Vasodilation caused by selective COX-2 inhibitors was attenuated in genetically obese Zucker rats because the production of the vasodilator PGE2 promoted by COX-2 in the endothelium was attenuated [84]. COX-2 inhibition can thus improve vasodilation over a limited range (Fig. 3).

Voltage-dependent K^+^ (Kv) channels regulate membrane resting potential and vascular tone. The opening of smooth muscle Kv channels results in hyperpolarization and vasodilation [85]. Dapagliflozin activates Kv channels by directly activating protein kinase G (PKG) independent of guanylyl cyclase, resulting in endothelial-independent vascular smooth muscle relaxation [86]. This kind of PKG/Kv channel signalling is effective and feasible [87]. TNF-α levels are elevated in T2DM and may impair insulin signalling and lead to insulin resistance [88]. Uthman et al. [89] reported that dapagliflozin and empagliflozin inhibited TNFα-induced ROS generation in human coronary arterial endothelial cells, and the subsequent decrease in NO consumption was responsible for improved blood vessel dilatation. An increase in L-arginine synthesis may indirectly improve coronary flow reserves by increasing NO synthesis. L-arginine is a substrate for nitric oxide synthase (NOS), which converts arginine to NO. An increase in L-arginine synthesis in the kidneys and an increase in NO bioavailability in response to SGLT2i were reported to increase coronary flow velocity reserve in an ob/ob − / − mouse model [90]. SGLT2i may act directly on cardiomyocyte NHE1 to reduce cytosolic Na^+^. Empagliflozin and canagliflozin significantly decreased coronary perfusion pressure in isolated C57 mouse hearts under constant-flow conditions, which was consistent with the dilation of coronary vessels [91].

### SGLT2i increase microvessel density in the heart

The microvascular complications of diabetes include a reduction in the density of arterioles on the surface of the heart and have also been described in animal models of diabetes [92, 93].

Some SGLT2i promote angiogenesis. For example, empagliflozin significantly reduced the loss of CD31 + microvessels and decreased the size of defects in zones of perfusion in diabetes model mice [46]. In the model, empagliflozin activated AMPK via an increased AMP/ATP ratio that then led to the failure of Drp1 recruitment to the mitochondria and weakened mitochondrial fission. The resulting reduction in ROS production alleviated cell senescence and decreased F-actin dissolution into G-actin, which contributed to cardiac microvascular endothelial cell migration and neovascularization.

Unfortunately, some SGLT2i inhibit angiogenesis. For example, canagliflozin inhibited the in vitro proliferation of human umbilical vein endothelial cells and the formation of blood vessels in allograft liver tumours [11]. Canagliflozin also inhibited angiogenesis in the lower limbs of mice with diabetes and lower limb ischaemia by inhibiting the secretion of vascular endothelial growth factor A by bone marrow-derived mesenchymal stem cells and reducing the proliferation and migration of mesenchymal stem cells [12].

## SGLT2i improve ventricular compliance and myocardial fibrosis

Myocardial fibrosis occurs in cardiomyopathy associated with diabetes, and it involves the accumulation of advanced glycation end products (AGEs) [94] and increased myocardial stiffness that interferes with ventricular diastole. Chronic hyperglycaemia is accompanied by the formation of AGEs that are produced by the nonenzymatic combination of glucose and proteins. Activation of the receptor for AGE (RAGE) by AGEs promotes the proliferation, function, and migration of cardiac fibroblasts, which exacerbates myocardial fibrosis and accelerates cardiac ageing [94–96]. AGEs, RAGE and downstream protein kinase C (PKC)-ζ and mitogen-activated protein kinase (MAPK) promote tissue fibrosis. Activation of the MAPK subfamily ERK is the most significant event in this process. After being activated, ERK translocates to the nucleus, where it influences transcription factors such as cAMP-response element binding protein, ETS domain-containing protein-1 and Y-box binding protein-1 to regulate cell proliferation, differentiation, and extracellular matrix accumulation [94, 97, 98]. Empagliflozin can inhibit the AGE/RAGE axis in the kidney, and it is believed that evidence in the heart will follow [99].

**Fig. 4:**
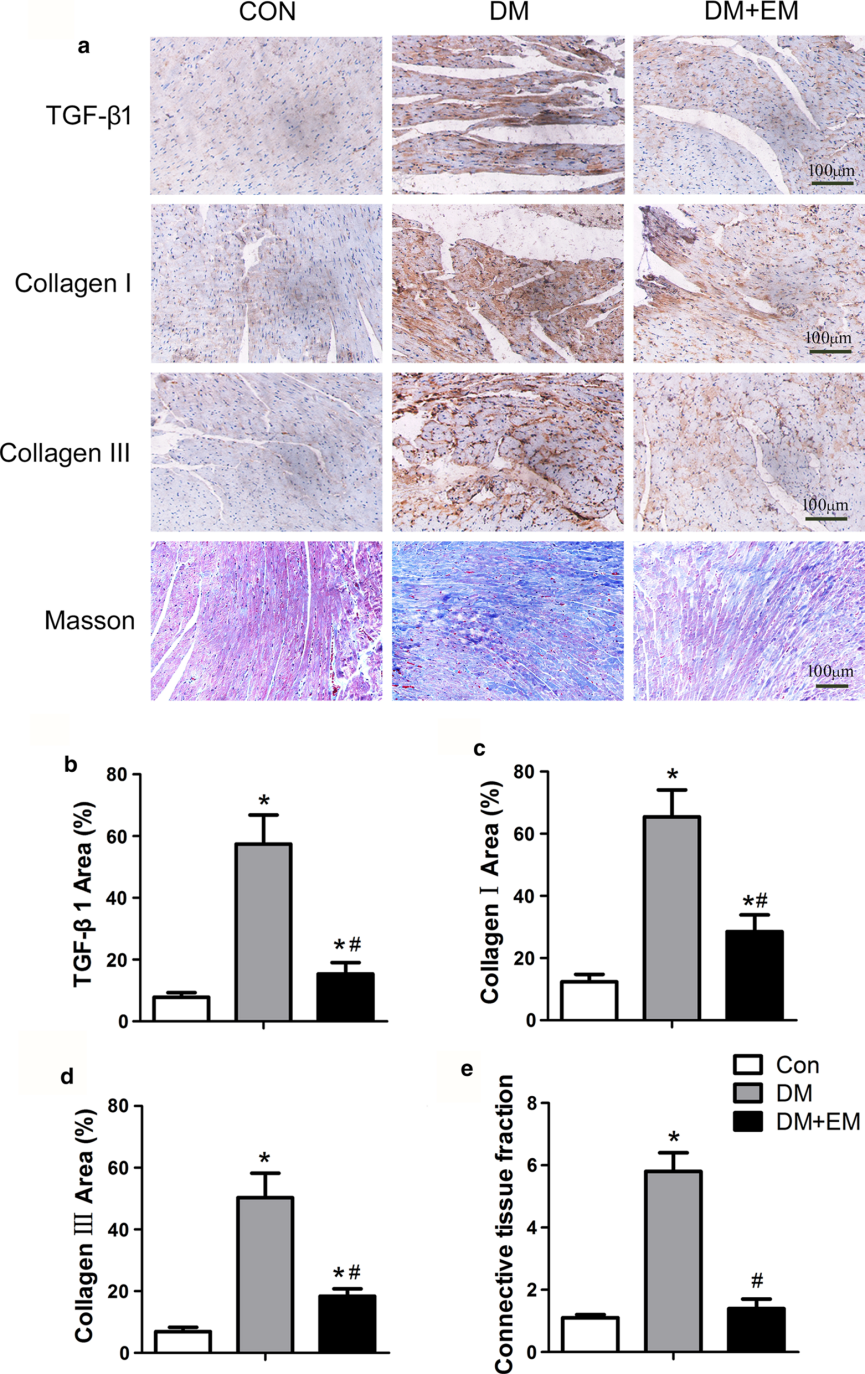
Histological data on the improvements in myocardial fibrosis by empagliflozin [102]. SGLT2 inhibitor empagliflozin played an important role in improving myocardial fibrosis of diabetic mice (genetic type 2 diabetes model) through reducing the expression of relevant signaling molecules and collagen. Compared with diabetic mice without empagliflozin treatment, it significantly reduced the expression of TGF-β1, p-Smad2, p-Smad3, collagen I, and collagen III. This kind of improvement represented the reduction in matrix accumulation and the betterment of ventricular compliance. Copyright 2019, Cardiovasc Diabetol.

**Fig. 5:**
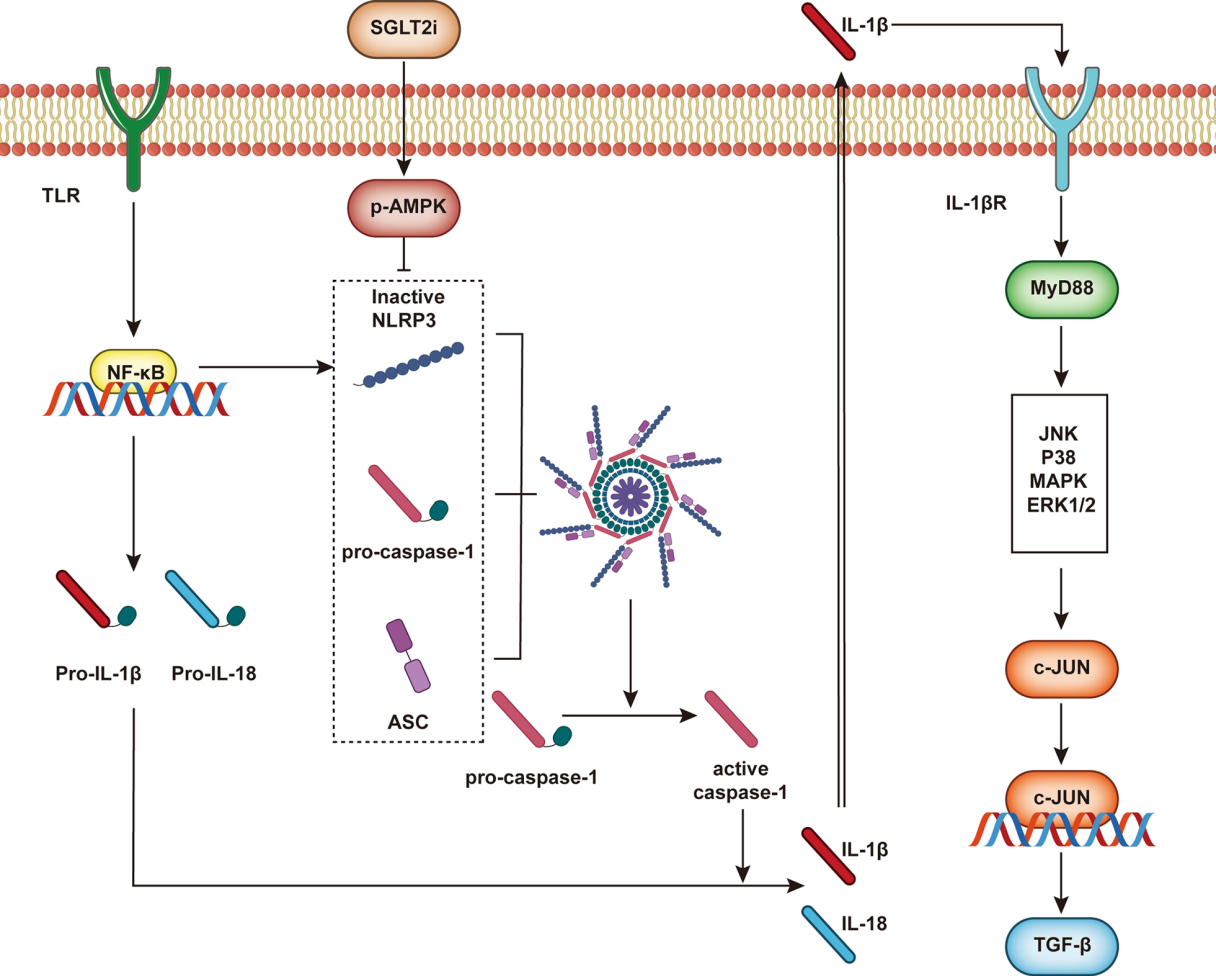
The SGLT2 inhibitor improves pyroptosis and reduces cardiac fibrosis. Activation of AMPK by the SGLT2 inhibitor decreases the expression of downstream NLRP3, Caspase-1 and ACS. Therefore, the upregulation of the NLRP3 inflammasome complex is inhibited, which reduces the transformation of procaspase-1 to active caspase-1 and the transformation of proIL-1β and proIL-18 to IL-1β and IL-18, respectively, to inhibit pyroptosis. As a result of the decrease in IL-1β, the activation of IL-1βR is correspondingly decreased, and the expression of TGF-β in the downstream signalling pathway is reduced, resulting in a relative reduction in fibrosis

Transforming growth factor-beta (TGF-β) also regulates tissue fibrosis via the SMAD protein, which is an intracellular effector downstream of the TGF-β receptor. Epithelial-myofibroblast transition and collagen expression are promoted by SMAD2 and SMAD3 and inhibited by SMAD7 [100, 101]. A recent study showed that myocardial fibrosis was improved by SGLT2i [102]. In a mouse model of type 2 diabetes, myocardial expression of collagen I and collagen III proteins and connective tissue fraction was increased compared with that in nondiabetic mice and was partially reversed by empagliflozin [102]. TGF-β/SMAD signalling was involved because, compared with that in untreated mice, the expression of TGF-β1, p-Smad2, and p-Smad3 in the heart tissue of mice treated with empagliflozin was significantly reduced (Fig. 4). Other studies have shown that blocking NOD-like receptor 3 (NLRP3) reduced myocardial fibrosis [103]. Interleukin (IL)-1β promotes *TGF-β* gene expression and promotes fibrosis through the TGF-β pathway. Other reasons involve nonpolymeric NLRP3 protein, but the mechanism is not very clear [104]. The TGF-β pathway is discussed in the pyroptosis section of this review (Fig. 5).

Diabetes increases the risk of myocardial infarction [60], and the infarct size is larger in patients with diabetes than in those without diabetes [105]. SGLT2i have been shown to improve ventricular remodelling after myocardial infarction and to alleviate cardiac fibrosis by modulating macrophage polarization. The mechanism is similar to that for atherosclerosis, as previously discussed. Studies have shown that in the postmortem myocardium, IL-10 can indirectly affect fibroblast activation by stimulating M2 macrophage polarization, thereby significantly reducing the level of collagen I, reducing the ratio of collagen I to collagen III in cardiac fibroblasts, and reducing collagen accumulation in the infarcted area of the mouse heart [106]. The increase in collagen I leads to increased fibril width and stiffness [107]. The effect of SGLT2i on this pathway was demonstrated by Lee et al. [108], who found that dapagliflozin induced macrophage polarization to the M2 phenotype and inhibited the M1 phenotype by stimulating signal transducer and activator of transcription 3 (STAT3) signalling in mice with myocardial infarction and attenuated the increase in collagen after infarction.

The effect of diabetes on ventricular diastolic dysfunction may be related to a reduction in cyclic guanosine monophosphate (cGMP) and a decrease in PKG activity because of increased nitrosative and oxidative stress and decreased bioavailability of NO [109, 110]. Oxidative and nitrosative stress caused by diabetes are discussed later in this review. PKG phosphorylates N2BA and N2B, the two main cardiac titin isoforms in the human left ventricle, resulting in a decrease in cardiomyofibrillar stiffness [111]. In human myocardial tissue from patients with heart failure with preserved ejection fraction (HFpEF) and ZDF obese rats, PKGIα was oxidized and was present as a dimer or polymer in the cardiomyocyte cell membrane. Empagliflozin decreased PKGIα oxidation and translocation of the reduced form into the cytoplasm of cardiomyocytes. Empagliflozin restored the damaged NO/soluble guanylate cyclase (sGC)/cGMP/PKG pathway in the HFpEF myocardium, significantly increased NO and cGMP concentrations, and increased sGC and PKGIα activity [112], which reduced cardiomyocyte stiffness. These changes were observed macroscopically. Echocardiography after intravenous injection of empagliflozin revealed significantly improved diastolic ventricular function in HFpEF rats and in humans [113].

## SGLT2i inhibit oxidative stress

Oxidative stress results from an imbalance in oxidative and antioxidative activity, and an excess of intermediate oxidative products damages cells. Hyperglycaemia and glucotoxicity result in oxidative stress in diabetes [114, 115], which is associated with lower antioxidant levels and higher oxidant levels than those in the absence of diabetes [116]. Therefore, a decrease in oxidative stress in the myocardium of diabetic patients would be key supporting evidence of their benefit in diabetic cardiomyopathy.

### SGLT2i reduce the production of oxidative intermediates

AMPK/Akt/endothelial nitric oxide synthase (eNOS) signalling may confer myocardial protection in diabetes. Oxidative stress and other pathologies uncouple eNOS, which is accompanied by abnormal electron transfer, and increase the production of ROS but not NO [117, 118], causing tissue damage. There is evidence that canagliflozin can activate eNOS in the myocardium and kidney. In an isoprenaline (ISO)-induced oxidative stress model, the phosphorylation of eNOS was significantly inhibited in the heart and kidney and was reversed by canagliflozin [119, 120]. Rescue was blocked by an AMPK inhibitor, which is consistent with the effect of AMPK/Akt signalling on canagliflozin activity. Another study reported that empagliflozin increased eNOS activity in the myocardium of obese mice fed a high-fat diet [121]. This evidence indicates that SGLT2i decrease oxidative stress, ROS and nitrate in tissues. The physiology of NO produced by NOS is well known. Inducible nitric oxide synthase (iNOS) differs from neurogenic nitric oxide synthase (nNOS) and eNOS and is associated with local inflammation [122, 123]. Compared with eNOS and nNOS, iNOS forms more superoxide and causes nitrative stress, which is effective for killing and inhibiting pathogens but also injures cells [124]. In an ISO-induced oxidative stress model, myocardial expression of iNOS was upregulated more than 3 times, but canagliflozin significantly reduced iNOS levels, which also decreased superoxide and nitrate [119]. Nox4 signalling has also been shown to induce iNOS [125]. Increased Nox4 activity has been associated with increased ROS production [126], and in an ISO-induced model of oxidative stress in mice, canagliflozin decreased Nox4 protein expression in the heart and kidney [119, 120].

### SGLT2i increases antioxidant activity

The nuclear factor erythroid factor 2 (Nrf2)/heme oxygenase (HO-1) pathway plays an important role in protecting cells when oxidative stress occurs in animal models. The activation of Nrf2 and HO-1 increased the expression of superoxide dismutase (SOD) and glutathione (GSH) and decreased the expression of malondialdehyde (MDA) compared with that in the control group without Nrf2 and HO-1 activation [127–129]. In a T2DM model, lipid hydroperoxide and MDA levels were significantly increased compared with those in the control group, and glutathione peroxidase (GSH-Px) and SOD were reduced. Empagliflozin could partially reverse the differences between the diabetes and control groups, indicating that oxidative stress was improved [102]. The mechanism of action involved activation of the Nrf2/HO-1 pathway. In other studies, canagliflozin had similar effects. Myocardial oxidative stress caused by ISO manifested as increased levels of MDA, advanced protein oxidation products, and myeloperoxidase and decreased levels of catalase, SOD, and GSH in the myocardium and plasma. The responses to ISO were reversed by canagliflozin treatment with a synchronous increase in Nrf2 [119].

Silencing information regulator 2 related enzyme 1 (SIRT1) regulates genes that attenuate oxidative stress in T2DM [130]. SIRT1 could decrease oxidative stress in diabetic cardiomyopathy [131, 132]. SGLT2i activate SIRT1 and its downstream signals [133, 134], which helps to explain how SGLT2i decrease oxidative stress in diabetic cardiomyopathy. The antioxidant effect induced by the activation of SIRT1 in T1DM diabetic cardiomyopathy mainly includes the SIRT1/Nrf2 signalling pathway [131], while in T2DM, it includes the Sirt1/forkhead box class O1 (FOXO1) signalling pathway [132]. Antioxidant activity mediated by SIRT1/FOXO1 was mediated by increased phosphorylation of SIRT1 and decreased acetylation of FOXO1. Deacetylation of FOXO1 by SIRT1 increases the transcription of cell cycle arrest genes, including those encoding antioxidant enzymes such as SOD2 [135].

## In diabetes patients, SGLT2i rescue cardiomyocytes from programmed cell death

Diabetes promotes programmed cells in the myocardium [136, 137], which results in decreased cardiac contractility, heart failure, and other complications. In the classic apoptosis pathway, the activation of caspase-3, caspase-6 and caspase-7 causes membrane blebbing, cell shrinkage, the formation of apoptotic bodies, and chromosomal DNA fragmentation. The pyroptosis pathway is mediated by caspases-1, -4, -5, and -11, which can cleave the cytosolic protein gasdermin D, and the latter can form large oligomeric pores in the inner layer of the plasma membrane and intracellular organelles to kill the cell [138].

### SGLT2i and myocardial apoptosis

Research on the inhibitory effect of SGLT2i on caspase-3 in the myocardium has begun. Trang et al. [139] showed that the levels of ERK1/2 and the proapoptotic gene Bax, which was promoted by ERK1/2, were increased, the level of pSTAT3, which can upregulate the expression of the antiapoptotic protein Bcl-2, was decreased, and the level of caspase-3 was increased in STZ-induced diabetic rat hearts. However, empagliflozin treatment changes these indicators to varying degrees [139]. Bax promotes mitochondrial outer membrane permeability, which results in the release of proapoptotic factors such as cytochrome c from the mitochondria into the cytoplasm to activate the caspase cascade [140]. Bcl-2 prevents the release of cytochrome c, thereby inhibiting the caspase cascade [141]. It is clear that inhibition of the ERK1/2 pathway and promotion of the STAT3 pathway by SGLT2i decrease cardiomyocyte apoptosis. In addition, high serum LPS originating from the gut microbiota contributes to myocardial inflammation and cell death in diabetes patients (see below). Koyani et al. [70] reported that empagliflozin reduced the LPS-induced increase in TNF-α levels associated with increased AMPK phosphorylation. TNF-α is known to activate caspase-3 via the caspase-12 cascade to induce cardiomyocyte apoptosis [142]. In animal models of heart I/R injury and hepatorenal syndrome, early administration of SGLT2i was reported to downregulate cleaved caspase-3 [143, 144], which is consistent with our expectations.

### SGLT2i and myocardial pyroptosis

Recent studies reported that SGLT2i inhibited caspase-1 and that the mechanism involved a pathway including AMPK/NLRP3/apoptosis-associated speck-like protein containing a CARD (ASC) [145–148]. Activation of Toll-like receptors (TLRs)/NF-κB and an increase in the transcription of inflammasome-related components, including inactive NLRP3, proIL-1β, and proIL-18. The oligomerization of inactive NLRP3, ASC, and procaspase-1 and the formation of an NLRP3-inflammasome complex [149] catalyse the conversion of procaspase-1 to caspase-1 and cause pyroptosis [150, 151]. The mRNA expression of NLRP3, ASC, IL-1β, and caspase-1 in cardiomyocytes did not increase significantly in wild-type mice but did so in the myocardia of type 2 diabetic (BTBR ob/ob) mice [152]. Dapagliflozin was not associated with the expression of NLRP3, ASC, IL-1β, or caspase-1 in normal wild-type mice but did reverse these factors in BTBR mice. Researchers then investigated the detailed mechanism of these changes. LPS increased the mRNA expression of NLRP3 and caspase-1 and decreased the P-AMPK/total-AMPK ratio in cardiac fibroblasts in wild-type and BTBR mice. Preincubation of cardiac fibroblasts with dapagliflozin attenuated the changes in the mRNA expression of NLRP3 and caspase-1 and the ratio of P-AMPK/total AMPK induced by LPS in wild-type and BTBR mice [152]. This finding suggests that the effectiveness of dapagliflozin was associated with AMPK, which was also reported in Chen et al. [153] (Fig. 5).

## SGLT2i and autophagy in the myocardium

Autophagy digests long-lived proteins and cytoplasmic organelles to meet the metabolic needs of the cell and the renewal of certain organelles [154, 155]. Autophagy imbalance and disruption occur in diabetes. Acute induction of autophagy may be beneficial, but persistent autophagy induction may be harmful [156, 157].

The effects of SGLT2i on autophagy have been studied in diabetic cardiomyopathy. Empagliflozin has been reported to increase autophagy in the atrial tissue of ZDF rats. This treatment increased the microtubule-associated protein light chain 3 (LC3) II/I ratio and decreased the protein expression of p62 in the atrial tissues of ZDF rats [25]. Li et al. [158] reported that empagliflozin decreased the protein expression of CD36, which was associated with an increase in p-AMPK, which activated the AMPK/Unc-51-like kinase 1 (Ulk1)/Beclin1 pathway, increased Ulk1 and Beclin1 expression, and promoted autophagy. This process in liver cancer cell experiments does not seem to involve mammalian target of rapamycin (mTOR). However, dapagliflozin decreased p-mTOR/mTOR in rat colitis model cells, suggesting that the inhibition of mTOR by AMPK [159] is involved in AMPK-mediated enhancement of autophagy [160]. The difference might be related to nutritional status in the two models because AMPK activates Ulk1 to promote autophagy during glucose starvation. In the presence of adequate nutrients, high mTOR activity phosphorylates Ulk1 at Ser 757, which disrupts the interaction between Ulk1 and AMPK to prevent Ulk1 activation [161].

SGLT2i do not enhance autophagy unilaterally. Jiang et al. [162] reported that in T1DM or T2DM and myocardial infarction, cardiomyocyte survival benefitted from the inhibition of enhanced autophagy by empagliflozin, which depended on the downregulation of NHE1 and NHE1-related genes that induce autophagy, such as *Beclin 1* and *autophagy-related protein 5*. In fact, the inhibitory effect of SGLT2i on NHE1 has been widely demonstrated [163], but it is not clear whether Beclin1 is a downstream target of NHE1. Deng et al. [164] found that Beclin1 but not NHE1 was targeted by empagliflozin. Empagliflozin was shown to inhibit the increase in autophagy caused by myocardial infarction with acute hyperglycaemia. Empagliflozin can also reverse the increase in the autophagy-related protein LC3II/I and decrease in P62 in cardiomyocytes induced by Tat-beclin1. This difference may be related to the difference in mouse models; the former model is a T2DM model with myocardial infarction, and the latter model is a nondiabetic myocardial infarction model with acute hyperglycaemia. However, these results show that the regulatory effect of empagliflozin on autophagy involves maintaining a balance, rather than unilaterally enhancing or weakening autophagy.

## SGLT2i reverse ER stress in diabetic cardiomyopathy

ER stress involves disturbances in Ca^2+^ or redox balance and the accumulation of misfolded or unfolded proteins that cannot be processed, which initiates an unfolded protein response (UPR) that leads to apoptosis [165]. The UPR involves three transducers: protein kinase RNA-like endoplasmic reticulum kinase (PERK), activated transcription factor 6 (ATF-6) and inositol-requiring protein-1α (IRE1α). The transducers bind to the ER chaperone glucose-regulated protein 78 (GRP78), and as unfolded proteins accumulate, GRP78 leaves the transducer and is involved in processing the accumulated proteins [166]. This leads to the activation of these three sensors and subsequent lethal effects [167]. Currently, the correlation between diabetes and ER stress has been well established [168].

PERK/eIF2α/ATF4/C/EBP-homologous protein (CHOP) are signalling pathways involved in ER stress. CHOP is a transcription factor that downregulates BCL2, BCL-XL, and MCL-1 expression and upregulates Bim, Bak and Bax expression. CHOP also upregulates the expression of the *pseudokinase tribbles homologue 3* gene, which was shown to weaken the inhibition of Caspse-9 and Caspase-3 expression by AKT [169–171].

**Fig. 6:**
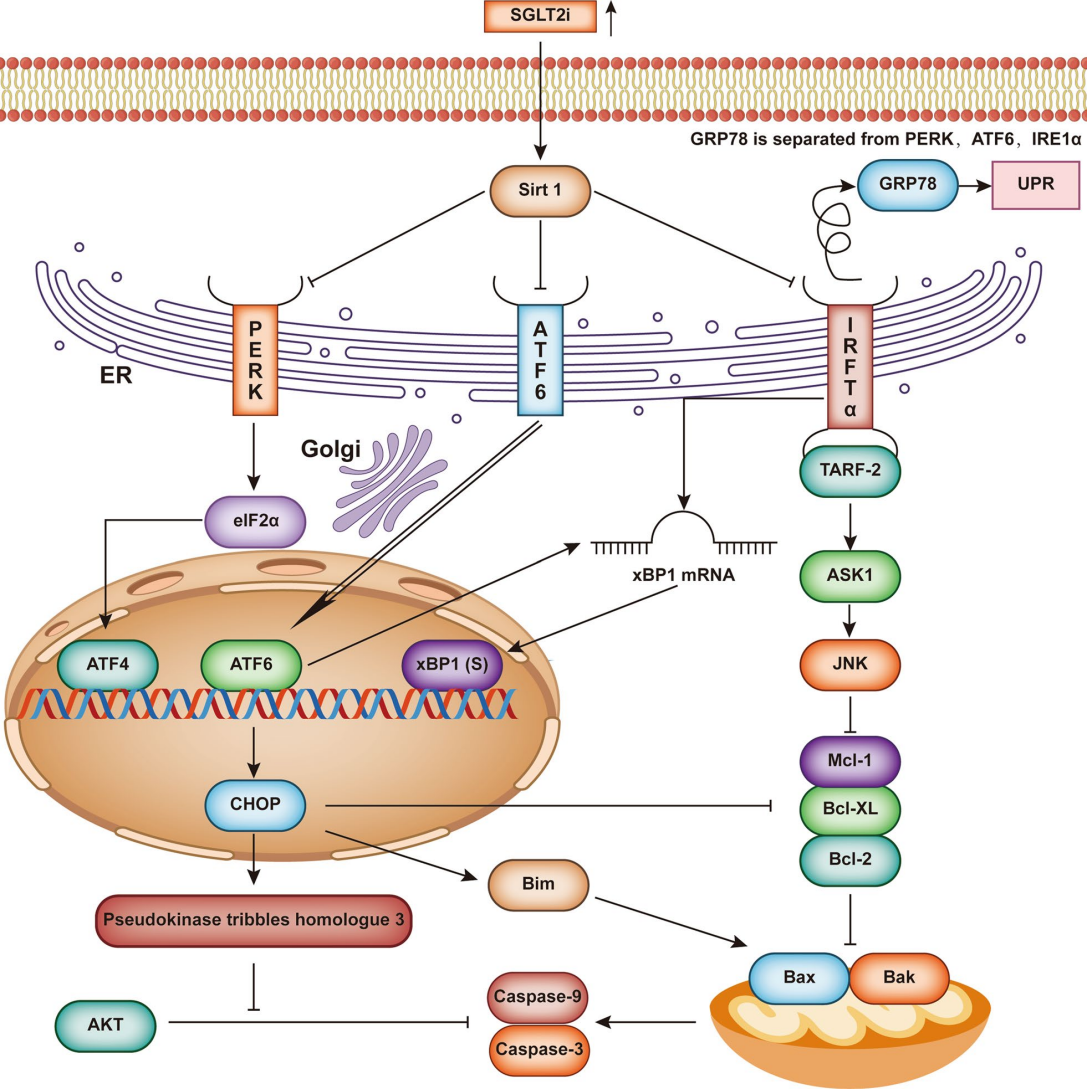
The SGLT2 inhibitor improves ER stress. SGLT2 inhibitor treatment inhibits the loss of GRP78 from transducers by activating Sirt1, thus inhibiting the development of ER stress. The downstream signalling pathways of PERK, ATF6 and IRE1α can induce the transcription of CHOP. CHOP upregulates the expression of the pseudokinase tribbles homologue 3 gene and weakens the inhibitory effect of AKT on the expression of caspase-9 and caspase-3. On the other hand, CHOP promotes the expression of Bax and Bak and inhibits the expression of Bcl-2, Bcl-XL and MCL-1. IRE1α also promotes apoptosis by sequentially activating ASK1 and JNK

SGLT2i ameliorate ER stress in animal models induced by heart-pressure overload or I/R injury [172, 173]. In these ER stress models induced by heart pressure overload or I/R injury, dapagliflozin and empagliflozin inhibited the increase in p-PERK and its downstream molecules associated with ER stress due to pressure overload by activating SIRT1 and preventing GRP78 detachment [172, 173]. Cell death was also significantly weakened. Dapagliflozin could significantly reduce GRP78, PERK, eIF-2α, ATF-4, and CHOP expression in the myocardium in an ER stress model induced by doxorubicin [174] (Fig. 6). SGLT2i have been shown to inhibit ER stress through similar downstream pathways in various organs. Ipraglifilozin inhibited the increase in GRP78 and PERK and their downstream signalling molecules associated with ectopic lipid deposition in mouse kidneys [175].

## SGLT2i improve diabetic cardiomyopathy by regulating the intestinal microbiota

The impact of intestinal flora imbalance on extraintestinal organs has received increasing attention in recent years, and the heart is no exception. The damaged intestinal wall can lead to the entry of intestinal bacteria into the circulation, causing inflammatory responses in multiple organs [176]. After entering the circulation, LPS from gram-negative bacteria is recognized by TLRs on the surface of immune cells and induces the release of proinflammatory cytokines [177]. Continuous infusion of low-dose LPS to mimic metabolic endotoxaemia leads to obesity, insulin resistance, T2DM, and atherosclerosis [178]. The metabolism of cholesterol and lipids by the gut microbiota affects the development of atherosclerotic plaques [179], and bacterial metabolites, such as trimethylamine N-oxide (TMAO), were shown to lead to myocardial fibrosis, abnormal metabolism, impaired endothelial function, and heart failure following the activation of various signalling pathways [180]. These pathological changes are consistent with the development of diabetic cardiomyopathy.

Dapagliflozin has been associated with changes in the gut microbiota, the function of extraintestinal blood vessels, and improvements in cardiovascular dysfunction caused by diabetes. Eight weeks of dapagliflozin treatment significantly increased *Akkermansia muciniphila* in the gut microbiota and improved generalized vascular dysfunction in mice with T2DM [181]. An increase in the abundance of *A. muciniphila* and improvements in glucose tolerance and blood glucose levels have been confirmed and correlated with induction of Foxp3-positive regulatory T cells [182]. An increase in *A. muciniphila* was also shown to reduce endotoxaemia and inflammation and prevent atherosclerosis by inducing the expression of tight junction proteins in the gut [183]. In addition, short-chain fatty acids (SCFAs) have been shown to thicken the mucin layer and strengthen the intestinal barrier by stimulating the release of IL-22 from lymphocytes, which prevents endotoxins and LPS from entering the body [184, 185]. Luseogliflozin increased the abundance of *Syntrophothermus lipocalidus*, family Syntrophomonadaceae, *Parabacteroidesdistasonis distasonis*, and genus Anaerotignum, which produce SCFAs [186]. Empagliflozin has been associated with an increase in the population of SCFA-producing bacteria and improvements in diabetes and cardiovascular function [187]. Roseburia, Eubacterium, and Faecalibacterium species were increased by empagliflozin treatment, and harmful bacteria, including Escherichia and Shigella, were decreased.

SGLT1 is the predominant receptor in the gut compared with SGLT2 [188]. Studies of the dual SGLT1/2 inhibitor canagliflozin and the SGLT1 inhibitor SGL5213 showed that these agents reversed the expansion of Firmicutes and contraction of Bacteroidetes in the gut microbiota of mice with adenine-induced renal failure [189, 190]. Increases in SCFAs such as acetate, butyrate, and propionate in the caecum and decreases in plasma TMAO levels were also found in a renal failure mouse model after treatment. These findings suggest that inhibition of SGLT1 reduces glucose uptake, while unabsorbed glucose temporarily reaches the lower small intestine [191], resulting in an altered glucose load and a possibly altered gut microbiota composition.

## SGLT2i in clinical treatment

The goal of basic research is to support clinical use. We have reviewed animal and clinical trials that evaluated the therapeutic effect of SGLT2i. The results of some animal trials support the performance of clinical trials. Here, we show some representative clinical evidence in response to the results of theoretical research (Table 2).

**Table 2:** Effect of SGLT2i in clinical treatment

Drugs	Type	Object	Follow-up period	Effect of outcome
Multiple SGLT2i	Clinical trial	77 first heart transplant recipients (37 patients with diabetes)	At least 6 months before surgery and 12 months after surgery	Reduce myocardial triglyceride accumulation [1]
Empagliflozin	Randomized controlled trial	97 participants with T2DM and coronary artery disease (CAD)	6 months	Reduce LVM indexed to body surface area [2]
Dapagliflozin	Randomized controlled trial	66 patients with T2DM and LVH	12 months	Reduce absolute LVM [3]
Dapagliflozin	Randomized controlled trial	97 patients with T2DM and atherosclerotic disease	12 weeks	Increase FMD [4]
Dapagliflozin	Randomized controlled trial	16 patients with T2DM and stable coronary artery disease	4 weeks	Increase MFR [5]
Dapagliflozin	Clinical trial	59 patients with T2DM	6 weeks	Improve vascular remodelling [6]
Multiple SGLT2i	Observational study	583 diabetic AMI patients treated with percutaneous coronary intervention (PCI)	The use of SGLT2i started at least 3 months before hospitalization	Reduce infarct size after AMI [7]
Empagliflozin	Clinical trial	1549 patients with T2DM	104 weeks	Reduce blood uric acid concentration [8]
Dapagliflozin	Clinical trial	3119 patients with heart failure	12 months	Reduce blood uric acid concentration [9]
Canagliflozin	Clinical trial	2313 patients with T2DM	26 weeks	Reduce blood uric acid concentration [10]
Dapagliflozin	Randomized controlled trial	44 patients with T2DM	12 weeks	Did not change the composition of the gut flora [11]
Multiple SGLT2i	Meta-analysis	38,335 patients with type 2 diabetes	Median follow-up duration was 1.8 years	Reduce the risk of AF and AFL [12]
Multiple SGLT2i	Meta-analysis	1831 patients with acute heart failure with and without T2DM	Ranged from 60 days to 9 months	Reduce the risk of rehospitalization for heart failure and improve KCCQ score [13]
Multiple SGLT2i	Meta-analysis	10978 patients with T2DM with or without chronic heart failure	Ranged from 14 days to 1 year	Reduce NT-proBNP concentrations and improve cardiac diastolic function and LVEF [14]

First, SGLT2i improves myocardial metabolism in diabetic patients. SGLT2i (dapagliflozin, empagliflozin, canagliflozin) reduce the expression of PPAR-γ in the hearts of patients. Similar to the mechanism described above, this effect can reduce the accumulation of fatty acids in myocardial cells [192], which is exactly what happened.

Empagliflozin is encouraging in terms of improving ventricular remodelling in diabetic patients. For example, in a clinical randomized controlled trial, the SGLT2i empagliflozin significantly reduced the left ventricular mass relative to body surface area [193]. Similarly, a randomized controlled trial of dapagliflozin demonstrated a significant reduction in left ventricular mass (LVM) in patients with T2DM and left ventricular hypertrophy (LVH) [194].

In diabetes patients with atherosclerotic disease, endothelial changes occur in the microvasculature and macrovasculature, and 12 weeks of dapagliflozin treatment resulted in a significant increase in FMD [195]. Another study demonstrated that 4 weeks of dapagliflozin increased myocardial flow reserve (MFR) in patients with stable coronary artery disease and T2DM [196]. In addition, clinical studies have confirmed that dapagliflozin can prevent microcirculation remodelling in diabetes patients and can decrease the stiffness of large blood vessels [197]. T2DM patients with acute myocardial infarction (AMI) who had received long-term treatment with SGLT2i before admission had smaller infarct sizes and lower inflammatory markers than those receiving other oral hypoglycaemic agents [198].

The effectiveness of SGLT2i for improving hyperuricaemia has been clinically verified. Dapagliflozin, empagliflozin, and canagliflozin have been shown to promote uric acid excretion in patients with or without diabetes [199–201]. This evidence shows that SGLT2i maintain cardiovascular function and reduce the risk of cardiovascular events.

The regulation of intestinal flora by SGLT2i is less clear. In a 12 week double-blind randomized trial, significant changes in gut microbiota diversity or composition were not observed in T2DM patients after dapagliflozin treatment [202]. The results may have been influenced by the administration of other drugs, including previous metformin monotherapy. In addition, the doses of dapagliflozin that are appropriate for treating diabetes may not be appropriate for regulating the gut microbiota. Additional studies of the impact of SGLT2i on the human intestinal microbiota are needed.

Several meta-analyses have provided more extensive and persuasive evidence that SGLT2i improve diabetes-induced cardiac insufficiency. T2DM increases the risk of atrial fibrillation (AF) and atrial flutter (AFL), and dapagliflozin significantly reduces AF and AFL [203]. Treatment of patients with AHF with SGLT2 inhibitors reduced the risk of rehospitalization due to heart failure and improved Kansas City Cardiomyopathy Questionnaire (KCCQ) scores [204]. SGLT2i improved diabetes-associated cardiac structure and function. A meta-analysis showed that SGLT2i partially reduced plasma NT-proBNP concentrations and improved cardiac diastolic function. However, SGLT2i improve left ventricular ejection fraction (LVEF) only in heart failure with reduced ejection fraction (HFrEF) in stage C heart failure [205]. On the other hand, in patients with HFpEF, especially those with stage A-B heart failure, the effect of SGLT2i treatment is not significant.

## Current challenges and future therapeutic strategies

SGLT2i are safe, well-tolerated drugs. They can improve all glycaemic parameters and have some additional benefits, such as weight and BP reductions, low risk of hypoglycaemia, improvements in β-cell function and insulin sensitivity, and reductions in macrovascular and microvascular events [206]. However, there are still many challenges to be solved in clinical practice. These challenges include but are not limited to decreased blood pressure, urinary and genital tract infections, amputation, ketoacidosis, kidney injury and fracture. Reduced blood pressure in SGLT2i users is understandable, which is caused by insufficient circulating blood volume due to osmotic diuresis. Meta-analyses showed that SGLT2i induced an average reduction in systolic/diastolic BP of 3.62/1.70 mmHg in 24 h ambulatory blood pressure [207]. However, the use of SGLT2i alone was very unlikely to cause postural hypotension. The use of SGLT2i has been reported to increase the risk of urinary and genital tract infections by more than 3 times [208]. The main reason may be related to the increase in urine sugar concentration. As previously mentioned, canagliflozin caused twice the risk of lower limb amputation compared to the control, possibly due to the inhibition of angiogenesis [209]. An increased incidence of ketoacidosis has been reported in SGLT2i users [210]. The prevailing belief is that this outcome is associated with glucose loss, increased hyperglucagonemia, constant or decreased insulin levels, mild infections, and decreased blood volume. This mechanism may induce euglycaemic diabetic ketoacidosis. The evidence that SGLT2i may cause acute renal injury is available [211], and the associated factors include circulatory failure, increased uric acid in urine, and multiple drug use [212, 213]. However, this treatment is not recognized as an inducer of acute renal injury, and several clinical trials have confirmed that SGLT2i have positive effects on kidney function [214]. In assessing the increased risk of fracture associated with SGLT2i, canagliflozin was directly associated with bone mineral density loss, especially in the hip. Dapagliflozin was associated with fractures unrelated to osteoporosis, mainly due to an increased risk of falls caused by fluctuations in blood pressure and hypoglycaemia caused by the combined use of hypoglycaemic drugs. However, empagliflozin was considered safe [215]. In addition, there are still some unresolved questions. The limited clinic evidence showed that SGLT2i improves the concentration of circulating proteins in plasma, serum or urine that are known to have beneficial effects on the heart [216]. These proteins include insulin-like growth factor-binding protein 1, transferrin receptor protein 1, erythropoietin and so on. But the exact target and signalling pathway of SGLT2i in clinic treatment are still not fully understood. The optimal dose of SGLT2i for diabetic cardiomyopathy and whether this dose is consistent with the dose for diabetic treatment still need experimental verification [217, 218].

In light of the current challenges associated with SGLT2i, we reviewed some of the guidelines and provided some insight into future strategies for the use of SGLT2i [218]. The use of SGLT2i is strongly recommended in patients with HFrEF and with or without T2DM, patients with T2DM and coronary heart disease, patients with T2DM were over 50 years old who had risk factors associated with coronary heart disease, and patients with albuminuric renal disease with or without T2DM. The benefits of SGLT2i on these patients have been well documented and reduce the risk of HF hospitalization. Moreover, we may avoid some possible complications by individualized medications and appropriate periodic examinations. For patients with suspected ketoacidosis symptoms such as nausea, vomiting, abdominal pain, and dyspnoea during SGLT2i use, blood and urine ketone body analysis should be performed in time. Patients who use SGLT2i should be advised to pay attention to perineal hygiene. For patients with urinary tract infection during medication use, SGLT2i administration should be paused, and anti-infective therapy should be given. SGLT2i should be discontinued in patients with recurrent urinary tract infections. Patients taking SGLT2i and diuretics together need to monitor their blood pressure. Canagliflozin is contraindicated in patients with lower extremity vascular stenosis or with osteoporosis. For renal insufficiency, patients with glomerular filtration rates less than 30 mL/min/1.73 m^2^ should be treated with low-dose SGLT2i or not treated with SGLT2i.

## Conclusions

In this review, we mainly focused on basic studies to elucidate the pharmacological mechanism of SGLT2i and related signalling pathways or targets for the treatment of diabetic cardiomyopathy. Studies have shown that SGLT2i can improve diabetic cardiomyopathy by improving myocardial metabolism, restoring mitochondrial function, improving microcirculatory disorders, reducing myocardial fibrosis, reducing oxidative stress, inhibiting programmed death, regulating autophagy, inhibiting ER stress and regulating the intestinal flora. In addition, we present representative clinical trials demonstrating the beneficial effects of SGLT2i in clinical use. Finally, we briefly discuss the current challenges and possible future strategies for the utilization of SGLT2i.

Our aim was to arouse more attention from readers through this review to further explore the potential mechanisms and targets of SGLT2i in the treatment of diabetic cardiomyopathy in the clinic and lay a solid foundation for the reasonable use of SGLT2i; SGLT2i can save more patients with diabetic cardiomyopathy. In addition, these findings could help in the development of new drugs for diabetic cardiomyopathy based on relevant signals or therapeutic targets.

## Data Availability

The data used to support the findings of this study are included within the article.

## Abbreviations

ACE: Angiotensin converting enzyme
AT1R: Angiotensin type 1 receptor
AF: Atrial fibrillation
AFL: Atrial flutter
AGEs: Advanced glycation end products
AMI: Acute myocardial infarction
AMPK: AMP-activated protein kinase
ASC: Apoptosis-associated speck-like protein containing a CARD
ATF-6: Transcription factor 6
β-OHB: Beta-hydroxybutyrate
cGMP: Cyclic guanosine monophosphate
CHOP: C/EBP-homologous protein
COX-2: Cyclooxygenase 2
CPT: Carnitine palmitoyltransferase
Drp1: Dynamin-related protein 1
EP4: E-type prostaglandin receptor 4
eNOS: Endothelial nitric oxide synthase
ER: Endoplasmic reticulum
ERK1/2: Extracellular signal-regulated kinase 1/2
ETC: Electron transport chain
FMD: Flow-mediated dilation
FOXO1: Forkhead box class O1
GLUT: Glucose transporter
GRP78: Glucose-regulated protein 78
GSH: Glutathione
GSH-Px: Glutathione peroxidase
HFpEF: Heart failure with preserved ejection fraction
HFrEF: Heart failure with reduced ejection fraction
HO-1: Heme oxygenase
IL: Interleukin
iNOS: Inducible nitric oxide synthase
I/R: Ischaemia/reperfusion
IRE1α: Inositol-requiring protein-1α
ISO: Isoprenaline
KCCQ: Kansas City Cardiomyopathy Questionnaire
Kv: Voltage-dependent K^+^
LC3: Microtubule-associated protein light chain 3
LPS: Lipopolysaccharide
LVEF: Left ventricular ejection fraction
LVH: Left ventricular hypertrophy
LVM: Left ventricular mass
MAPK: Mitogen-activated protein kinase
MDA: Malondialdehyde
MFNs: Mitofusins
MFR: Myocardial flow reserve
mTOR: Mammalian target of rapamycin
mtTFA: Mitochondrial transcription factor A
NADPH: Nicotinamide adenine dinucleotide phosphate
NHE1: Na^+^/H^+^ exchanger-1
NLRP3: NOD-like receptor 3
nNOS: Neurogenic nitric oxide synthase
NOS: Nitric oxide synthase
Nox4: NADPH oxidase isoform 4
NRF: Nuclear respiratory factors
Nrf2: Nuclear factor erythroid factor 2
OPA1: Optic atrophy 1
PERK: RNA-like endoplasmic reticulum kinase
PGC-1α: Peroxisome proliferator-activated receptor γ coactivator-1α
PKC: Protein kinase C
PKG: Protein kinase G
PPARs: Peroxisome proliferator-activated receptors
RAGE: Receptor for AGE
ROS: Reactive oxygen species
SCFAs: Short-chain fatty acids
sGC: Soluble guanylate cyclase
SGLT2is: Sodium-glucose cotransporter 2 inhibitors
SIRT1: Silencing information regulator 2 related enzyme 1
SOD: Superoxide dismutase
STAT3: Signal transducer and activator of transcription 3
STZ: Streptozotocin
T1DM: Type 1 diabetes mellitus
T2DM: Type 2 diabetes mellitus
TC: Total cholesterol
TG: Triglycerides
TGF-β: Growth factor-beta
TLRs: Toll-like receptors
TMAO: Trimethylamine N-oxide
TNF-α: Tumour necrosis factor-α
Ulk1: Unc-51-like kinase 1
UPR: Unfolded protein response
VCAM-1: Vascular cell adhesion molecule-1

## References

[1] Ahmed AM (2002). History of diabetes mellitus. Saudi Med J.

[2] Zheng Y, Ley SH, Hu FB (2018). Global aetiology and epidemiology of type 2 diabetes mellitus and its complications. Nat Rev Endocrinol.

[3] Jia G, Whaley-Connell A, Sowers JR (2018). Diabetic cardiomyopathy: a hyperglycaemia- and insulin-resistance-induced heart disease. Diabetologia.

[4] Isfort M, Stevens SC, Schaffer S, Jong CJ, Wold LE (2014). Metabolic dysfunction in diabetic cardiomyopathy. Heart Fail Rev.

[5] Bugger H, Abel ED (2014). Molecular mechanisms of diabetic cardiomyopathy. Diabetologia.

[6] Holscher ME, Bode C, Bugger H (2016). Diabetic cardiomyopathy: does the type of diabetes matter?. Int J Mol Sci.

[7] Reifsnider OS, Kansal AR, Gandhi PK, Cragin L, Brand SB, Pfarr E (2021). Cost-effectiveness of empagliflozin versus canagliflozin, dapagliflozin, or standard of care in patients with type 2 diabetes and established cardiovascular disease. BMJ Open Diabetes Res Care.

[8] Cefalo CMA, Cinti F, Moffa S, Impronta F, Sorice GP, Mezza T (2019). Sotagliflozin, the first dual SGLT inhibitor: current outlook and perspectives. Cardiovasc Diabetol.

[9] Isaji M (2011). SGLT2 inhibitors: molecular design and potential differences in effect. Kidney Int Suppl.

[10] Saisho Y (2020). SGLT2 inhibitors: the star in the treatment of type 2 diabetes?. Diseases.

[11] Kaji K, Nishimura N, Seki K, Sato S, Saikawa S, Nakanishi K (2018). Sodium glucose cotransporter 2 inhibitor canagliflozin attenuates liver cancer cell growth and angiogenic activity by inhibiting glucose uptake. Int J Cancer.

[12] Lin Y, Nan J, Shen J, Lv X, Chen X, Lu X (2020). Canagliflozin impairs blood reperfusion of ischaemic lower limb partially by inhibiting the retention and paracrine function of bone marrow derived mesenchymal stem cells. EBioMedicine.

[13] Kondo H, Akoumianakis I, Badi I, Akawi N, Kotanidis CP, Polkinghorne M (2021). Effects of canagliflozin on human myocardial redox signalling: clinical implications. Eur Heart J.

[14] Uthman L, Baartscheer A, Schumacher CA, Fiolet JWT, Kuschma MC, Hollmann MW (2018). Direct cardiac actions of sodium glucose cotransporter 2 inhibitors target pathogenic mechanisms underlying heart failure in diabetic patients. Front Physiol.

[15] Philippaert K, Kalyaanamoorthy S, Fatehi M, Long W, Soni S, Byrne NJ (2021). Cardiac late sodium channel current is a molecular target for the sodium/glucose cotransporter 2 inhibitor empagliflozin. Circulation.

[16] Amaral N, Okonko DO (2015). Metabolic abnormalities of the heart in type II diabetes. Diab Vasc Dis Res.

[17] Dakhili SAT, Greenwell AA, Ussher JR (2023). Pyruvate dehydrogenase complex and glucose oxidation as a therapeutic target in diabetic heart disease. J Lipid Atheroscler.

[18] Mizuno Y, Harada E, Nakagawa H, Morikawa Y, Shono M, Kugimiya F (2017). The diabetic heart utilizes ketone bodies as an energy source. Metabolism.

[19] Bertrand L, Auquier J, Renguet E, Angé M, Cumps J, Horman S (2020). Glucose transporters in cardiovascular system in health and disease. Pflugers Arch.

[20] Mustroph J, Lücht CM, Wagemann O, Sowa T, Hammer KP, Sag CM (2019). Empagliflozin enhances human and murine cardiomyocyte glucose uptake by increased expression of GLUT1. Diabetologia.

[21] Verma S, Rawat S, Ho KL, Wagg CS, Zhang L, Teoh H (2018). Empagliflozin increases cardiac energy production in diabetes: novel translational insights into the heart failure benefits of SGLT2 inhibitors. JACC Basic Transl Sci.

[22] Angelini A, Saha PK, Jain A, Jung SY, Mynatt RL, Pi X (2021). PHDs/CPT1B/VDAC1 axis regulates long-chain fatty acid oxidation in cardiomyocytes. Cell Rep.

[23] Oshima H, Miki T, Kuno A, Mizuno M, Sato T, Tanno M (2019). Empagliflozin, an SGLT2 inhibitor, reduced the mortality rate after acute myocardial infarction with modification of cardiac metabolomes and antioxidants in diabetic rats. J Pharmacol Exp Ther.

[24] Huang CC, Chou CA, Chen WY, Yang JL, Lee WC, Chen JB (2021). Empagliflozin ameliorates free fatty acid induced-lipotoxicity in renal proximal tubular cells via the PPARγ/CD36 pathway in obese mice. Int J Mol Sci.

[25] Aragon-Herrera A, Feijoo-Bandin S, Santiago MO, Barral L, Campos-Toimil M, Gil-Longo J (2019). Empagliflozin reduces the levels of CD36 and cardiotoxic lipids while improving autophagy in the hearts of Zucker diabetic fatty rats. Biochem Pharmacol.

[26] Pepino MY, Kuda O, Samovski D, Abumrad NA (2014). Structure-function of CD36 and importance of fatty acid signal transduction in fat metabolism. Annu Rev Nutr.

[27] Wang L, Cai Y, Jian L, Cheung CW, Zhang L, Xia Z (2021). Impact of peroxisome proliferator-activated receptor-α on diabetic cardiomyopathy. Cardiovasc Diabetol.

[28] Wei D, Liao L, Wang H, Zhang W, Wang T, Xu Z (2020). Canagliflozin ameliorates obesity by improving mitochondrial function and fatty acid oxidation via PPARα in vivo and in vitro. Life Sci.

[29] Yanai H, Yoshida H (2019). Beneficial effects of adiponectin on glucose and lipid metabolism and atherosclerotic progression: mechanisms and perspectives. Int J Mol Sci.

[30] Wu P, Wen W, Li J, Xu J, Zhao M, Chen H (2019). Systematic review and meta-analysis of randomized controlled trials on the effect of SGLT2 inhibitor on blood leptin and adiponectin level in patients with type 2 diabetes. Horm Metab Res.

[31] Krauss RM (2004). Lipids and lipoproteins in patients with type 2 diabetes. Diabetes Care.

[32] Liu L, Mu Y, Han W, Wang C (2014). Association of hypercholesterolemia and cardiac function evaluated by speckle tracking echocardiography in a rabbit model. Lipids Health Dis.

[33] Huang Y, Walker KE, Hanley F, Narula J, Houser SR, Tulenko TN (2004). Cardiac systolic and diastolic dysfunction after a cholesterol-rich diet. Circulation.

[34] Kim JH, Lee M, Kim SH, Kim SR, Lee BW, Kang ES (2019). Sodium-glucose cotransporter 2 inhibitors regulate ketone body metabolism via inter-organ crosstalk. Diabetes Obes Metab.

[35] Pietschner R, Kolwelter J, Bosch A, Striepe K, Jung S, Kannenkeril D (2021). Effect of empagliflozin on ketone bodies in patients with stable chronic heart failure. Cardiovasc Diabetol.

[36] Ferrannini E, Mark M, Mayoux E (2016). CV protection in the EMPA-REG OUTCOME trial: a “thrifty substrate” hypothesis. Diabetes Care.

[37] Santos-Gallego CG, Requena-Ibanez JA, San Antonio R, Ishikawa K, Watanabe S, Picatoste B (2019). Empagliflozin ameliorates adverse left ventricular remodeling in nondiabetic heart failure by enhancing myocardial energetics. J Am Coll Cardiol.

[38] Galloway CA, Yoon Y (2015). Mitochondrial dynamics in diabetic cardiomyopathy. Antioxid Redox Signal.

[39] Wu S, Lu Q, Ding Y, Wu Y, Qiu Y, Wang P (2019). Hyperglycemia-driven inhibition of AMP-activated protein kinase α2 induces diabetic cardiomyopathy by promoting mitochondria-associated endoplasmic reticulum membranes in vivo. Circulation.

[40] Zheng H, Zhu H, Liu X, Huang X, Huang A, Huang Y (2021). Mitophagy in diabetic cardiomyopathy: roles and mechanisms. Front Cell Dev Biol.

[41] Bereiter-Hahn J, Vöth M (1994). Dynamics of mitochondria in living cells: shape changes, dislocations, fusion, and fission of mitochondria. Microsc Res Tech.

[42] Zemirli N, Morel E, Molino D (2018). Mitochondrial dynamics in basal and stressful conditions. Int J Mol Sci.

[43] Yu T, Robotham JL, Yoon Y (2006). Increased production of reactive oxygen species in hyperglycemic conditions requires dynamic change of mitochondrial morphology. Proc Natl Acad Sci USA.

[44] Tanajak P, Sa-Nguanmoo P, Sivasinprasasn S, Thummasorn S, Siri-Angkul N, Chattipakorn SC (2018). Cardioprotection of dapagliflozin and vildagliptin in rats with cardiac ischemia-reperfusion injury. J Endocrinol.

[45] Chang CR, Blackstone C (2010). Dynamic regulation of mitochondrial fission through modification of the dynamin-related protein Drp1. Ann N Y Acad Sci.

[46] Zhou H, Wang S, Zhu P, Hu S, Chen Y, Ren J (2018). Empagliflozin rescues diabetic myocardial microvascular injury via AMPK-mediated inhibition of mitochondrial fission. Redox Biol.

[47] Tilokani L, Nagashima S, Paupe V, Prudent J (2018). Mitochondrial dynamics: overview of molecular mechanisms. Essays Biochem.

[48] Chen H, Detmer SA, Ewald AJ, Griffin EE, Fraser SE, Chan DC (2003). Mitofusins Mfn1 and Mfn2 coordinately regulate mitochondrial fusion and are essential for embryonic development. J Cell Biol.

[49] Sun S, Erchova I, Sengpiel F, Votruba M (2020). Opa1 deficiency leads to diminished mitochondrial bioenergetics with compensatory increased mitochondrial motility. Invest Ophthalmol Vis Sci.

[50] Durak A, Olgar Y, Degirmenci S, Akkus E, Tuncay E, Turan B (2018). A SGLT2 inhibitor dapagliflozin suppresses prolonged ventricular-repolarization through augmentation of mitochondrial function in insulin-resistant metabolic syndrome rats. Cardiovasc Diabetol.

[51] Shao Q, Meng L, Lee S, Tse G, Gong M, Zhang Z (2019). Empagliflozin, a sodium glucose co-transporter-2 inhibitor, alleviates atrial remodeling and improves mitochondrial function in high-fat diet/streptozotocin-induced diabetic rats. Cardiovasc Diabetol.

[52] Jornayvaz FR, Shulman GI (2010). Regulation of mitochondrial biogenesis. Essays Biochem.

[53] Scarpulla RC (2011). Metabolic control of mitochondrial biogenesis through the PGC-1 family regulatory network. Biochim Biophys Acta.

[54] Virbasius JV, Scarpulla RC (1994). Activation of the human mitochondrial transcription factor A gene by nuclear respiratory factors: a potential regulatory link between nuclear and mitochondrial gene expression in organelle biogenesis. Proc Natl Acad Sci USA.

[55] Irrcher I, Adhihetty PJ, Sheehan T, Joseph AM, Hood DA (2003). PPARgamma coactivator-1alpha expression during thyroid hormone- and contractile activity-induced mitochondrial adaptations. Am J Physiol Cell Physiol.

[56] Croston TL, Thapa D, Holden AA, Tveter KJ, Lewis SE, Shepherd DL (2014). Functional deficiencies of subsarcolemmal mitochondria in the type 2 diabetic human heart. Am J Physiol Heart Circ Physiol.

[57] Secker PF, Beneke S, Schlichenmaier N, Delp J, Gutbier S, Leist M (2018). Canagliflozin mediated dual inhibition of mitochondrial glutamate dehydrogenase and complex I: an off-target adverse effect. Cell Death Dis.

[58] Hu Y, Suarez J, Fricovsky E, Wang H, Scott BT, Trauger SA (2009). Increased enzymatic O-GlcNAcylation of mitochondrial proteins impairs mitochondrial function in cardiac myocytes exposed to high glucose. J Biol Chem.

[59] Hodrea J, Balogh DB, Hosszu A, Lenart L, Besztercei B, Koszegi S (2020). Reduced O-GlcNAcylation and tubular hypoxia contribute to the antifibrotic effect of SGLT2 inhibitor dapagliflozin in the diabetic kidney. Am J Physiol Renal Physiol.

[60] Huang D, Refaat M, Mohammedi K, Jayyousi A, Al Suwaidi J, Abi KC (2017). Macrovascular complications in patients with diabetes and prediabetes. Biomed Res Int.

[61] Kibel A, Selthofer-Relatic K, Drenjancevic I, Bacun T, Bosnjak I, Kibel D (2017). Coronary microvascular dysfunction in diabetes mellitus. J Int Med Res.

[62] Henning RJ (2018). Type-2 diabetes mellitus and cardiovascular disease. Future Cardiol.

[63] Poznyak AV, Litvinova L, Poggio P, Sukhorukov VN, Orekhov AN (2022). Effect of glucose levels on cardiovascular risk. Cells.

[64] Forrester SJ, Preston KJ, Cooper HA, Boyer MJ, Escoto KM, Poltronetti AJ (2020). Mitochondrial fission mediates endothelial inflammation. Hypertension.

[65] Gimbrone MA, García-Cardeña G (2016). Endothelial cell dysfunction and the pathobiology of atherosclerosis. Circ Res.

[66] Wang Q, Zhang M, Torres G, Wu S, Ouyang C, Xie Z (2017). Metformin suppresses diabetes-accelerated atherosclerosis via the inhibition of Drp1-mediated mitochondrial fission. Diabetes.

[67] Tian K, Xu Y, Sahebkar A, Xu S (2020). CD36 in atherosclerosis: pathophysiological mechanisms and therapeutic implications. Curr Atheroscler Rep.

[68] Blagov AV, Markin AM, Bogatyreva AI, Tolstik TV, Sukhorukov VN, Orekhov AN (2023). The role of macrophages in the pathogenesis of atherosclerosis. Cells.

[69] Pennig J, Scherrer P, Gissler MC, Anto-Michel N, Hoppe N, Füner L (2019). Glucose lowering by SGLT2-inhibitor empagliflozin accelerates atherosclerosis regression in hyperglycemic STZ-diabetic mice. Sci Rep.

[70] Koyani CN, Plastira I, Sourij H, Hallstrom S, Schmidt A, Rainer PP (2020). Empagliflozin protects heart from inflammation and energy depletion via AMPK activation. Pharmacol Res.

[71] Shan H, Bai X, Chen X (2008). Angiotensin II induces endothelial cell senescence via the activation of mitogen-activated protein kinases. Cell Biochem Funct.

[72] Khemais-Benkhiat S, Belcastro E, Idris-Khodja N, Park SH, Amoura L, Abbas M (2020). Angiotensin II-induced redox-sensitive SGLT1 and 2 expression promotes high glucose-induced endothelial cell senescence. J Cell Mol Med.

[73] Lee BY, Han JA, Im JS, Morrone A, Johung K, Goodwin EC (2006). Senescence-associated beta-galactosidase is lysosomal beta-galactosidase. Aging Cell.

[74] Lv Q, Meng XF, He FF, Chen S, Su H, Xiong J (2013). High serum uric acid and increased risk of type 2 diabetes: a systemic review and meta-analysis of prospective cohort studies. PLoS ONE.

[75] Katsiki N, Papanas N, Fonseca VA, Maltezos E, Mikhailidis DP (2013). Uric acid and diabetes: is there a link?. Curr Pharm Des.

[76] Albu A, Para I, Porojan M (2020). Uric acid and arterial stiffness. Ther Clin Risk Manag.

[77] Chino Y, Samukawa Y, Sakai S, Nakai Y, Yamaguchi J, Nakanishi T (2014). SGLT2 inhibitor lowers serum uric acid through alteration of uric acid transport activity in renal tubule by increased glycosuria. Biopharm Drug Dispos.

[78] Shah AS, Urbina EM (2017). Vascular and endothelial function in youth with type 2 diabetes mellitus. Curr Diab Rep.

[79] Gallinoro E, Paolisso P, Candreva A, Bermpeis K, Fabbricatore D, Esposito G (2021). Microvascular dysfunction in patients with type II diabetes mellitus: invasive assessment of absolute coronary blood flow and microvascular resistance reserve. Front Cardiovasc Med.

[80] Shigiyama F, Kumashiro N, Miyagi M, Ikehara K, Kanda E, Uchino H (2017). Effectiveness of dapagliflozin on vascular endothelial function and glycemic control in patients with early-stage type 2 diabetes mellitus: DEFENCE study. Cardiovasc Diabetol.

[81] Sancho P, Martin-Sanz P, Fabregat I (2011). Reciprocal regulation of NADPH oxidases and the cyclooxygenase-2 pathway. Free Radic Biol Med.

[82] Ardanaz N, Pagano PJ (2006). Hydrogen peroxide as a paracrine vascular mediator: regulation and signaling leading to dysfunction. Exp Biol Med.

[83] Oelze M, Kroller-Schon S, Welschof P, Jansen T, Hausding M, Mikhed Y (2014). The sodium-glucose co-transporter 2 inhibitor empagliflozin improves diabetes-induced vascular dysfunction in the streptozotocin diabetes rat model by interfering with oxidative stress and glucotoxicity. PLoS ONE.

[84] Santiago E, Martínez MP, Climent B, Muñoz M, Briones AM, Salaices M (2016). Augmented oxidative stress and preserved vasoconstriction induced by hydrogen peroxide in coronary arteries in obesity: role of COX-2. Br J Pharmacol.

[85] Hasan R, Jaggar JH (2018). K_V_ channel trafficking and control of vascular tone. Microcirculation.

[86] Li H, Shin SE, Seo MS, An JR, Choi IW, Jung WK (2018). The anti-diabetic drug dapagliflozin induces vasodilation via activation of PKG and Kv channels. Life Sci.

[87] Ko EA, Park WS, Firth AL, Kim N, Yuan JX, Han J (2010). Pathophysiology of voltage-gated K+ channels in vascular smooth muscle cells: modulation by protein kinases. Prog Biophys Mol Biol.

[88] Akash MSH, Rehman K, Liaqat A (2018). Tumor necrosis factor-alpha: role in development of insulin resistance and pathogenesis of type 2 diabetes mellitus. J Cell Biochem.

[89] Uthman L, Homayr A, Juni RP, Spin EL, Kerindongo R, Boomsma M (2019). Empagliflozin and dapagliflozin reduce ROS generation and restore NO bioavailability in tumor necrosis factor α-stimulated human coronary arterial endothelial cells. Cell Physiol Biochem.

[90] Adingupu DD, Göpel SO, Grönros J, Behrendt M, Sotak M, Miliotis T (2019). SGLT2 inhibition with empagliflozin improves coronary microvascular function and cardiac contractility in prediabetic ob/ob(–/–) mice. Cardiovasc Diabetol.

[91] Uthman L, Baartscheer A, Bleijlevens B, Schumacher CA, Fiolet JWT, Koeman A (2018). Class effects of SGLT2 inhibitors in mouse cardiomyocytes and hearts: inhibition of Na(+)/H(+) exchanger, lowering of cytosolic Na(+) and vasodilation. Diabetologia.

[92] Zeng H, He X, Hou X, Li L, Chen JX (2014). Apelin gene therapy increases myocardial vascular density and ameliorates diabetic cardiomyopathy via upregulation of sirtuin 3. Am J Physiol Heart Circ Physiol.

[93] Rawal S, Munasinghe PE, Shindikar A, Paulin J, Cameron V, Manning P (2017). Down-regulation of proangiogenic microRNA-126 and microRNA-132 are early modulators of diabetic cardiac microangiopathy. Cardiovasc Res.

[94] Zhao J, Randive R, Stewart JA (2014). Molecular mechanisms of AGE/RAGE-mediated fibrosis in the diabetic heart. World J Diabetes.

[95] Shamhart PE, Luther DJ, Adapala RK, Bryant JE, Petersen KA, Meszaros JG (2014). Hyperglycemia enhances function and differentiation of adult rat cardiac fibroblasts. Can J Physiol Pharmacol.

[96] Fang M, Wang J, Li S, Guo Y (2016). Advanced glycation end-products accelerate the cardiac aging process through the receptor for advanced glycation end-products/transforming growth factor-β-Smad signaling pathway in cardiac fibroblasts. Geriatr Gerontol Int.

[97] Gallo S, Vitacolonna A, Bonzano A, Comoglio P, Crepaldi T (2019). ERK: a key player in the pathophysiology of cardiac hypertrophy. Int J Mol Sci.

[98] Zhong X, Wang T, Zhang W, Wang M, Xie Y, Dai L (2022). ERK/RSK-mediated phosphorylation of Y-box binding protein-1 aggravates diabetic cardiomyopathy by suppressing its interaction with deubiquitinase OTUB1. J Biol Chem.

[99] Ojima A, Matsui T, Nishino Y, Nakamura N, Yamagishi S (2015). Empagliflozin, an inhibitor of sodium-glucose cotransporter 2 exerts anti-inflammatory and antifibrotic effects on experimental diabetic nephropathy partly by suppressing AGEs-receptor axis. Horm Metab Res.

[100] Xu F, Liu C, Zhou D, Zhang L (2016). TGF-β/SMAD pathway and its regulation in hepatic fibrosis. J Histochem Cytochem.

[101] Ma TT, Meng XM (2019). TGF-β/Smad and renal fibrosis. Adv Exp Med Biol.

[102] Li C, Zhang J, Xue M, Li X, Han F, Liu X (2019). SGLT2 inhibition with empagliflozin attenuates myocardial oxidative stress and fibrosis in diabetic mice heart. Cardiovasc Diabetol.

[103] Gao R, Shi H, Chang S, Gao Y, Li X, Lv C (2019). The selective NLRP3-inflammasome inhibitor MCC950 reduces myocardial fibrosis and improves cardiac remodeling in a mouse model of myocardial infarction. Int Immunopharmacol.

[104] Alyaseer AAA, de Lima MHS, Braga TT (2020). The role of NLRP3 inflammasome activation in the epithelial to mesenchymal transition process during the fibrosis. Front Immunol.

[105] Marso SP, Miller T, Rutherford BD, Gibbons RJ, Qureshi M, Kalynych A (2007). Comparison of myocardial reperfusion in patients undergoing percutaneous coronary intervention in ST-segment elevation acute myocardial infarction with versus without diabetes mellitus (from the EMERALD trial). Am J Cardiol.

[106] Jung M, Ma Y, Iyer RP, DeLeon-Pennell KY, Yabluchanskiy A, Garrett MR (2017). IL-10 improves cardiac remodeling after myocardial infarction by stimulating M2 macrophage polarization and fibroblast activation. Basic Res Cardiol.

[107] Li W, Chi N, Rathnayake RAC, Wang R (2021). Distinctive roles of fibrillar collagen I and collagen III in mediating fibroblast-matrix interaction: a nanoscopic study. Biochem Biophys Res Commun.

[108] Lee TM, Chang NC, Lin SZ (2017). Dapagliflozin, a selective SGLT2 inhibitor, attenuated cardiac fibrosis by regulating the macrophage polarization via STAT3 signaling in infarcted rat hearts. Free Radic Biol Med.

[109] Paulus WJ, Tschöpe C (2013). A novel paradigm for heart failure with preserved ejection fraction: comorbidities drive myocardial dysfunction and remodeling through coronary microvascular endothelial inflammation. J Am Coll Cardiol.

[110] van Heerebeek L, Hamdani N, Falcao-Pires I, Leite-Moreira AF, Begieneman MP, Bronzwaer JG (2012). Low myocardial protein kinase G activity in heart failure with preserved ejection fraction. Circulation.

[111] Krüger M, Kötter S, Grützner A, Lang P, Andresen C, Redfield MM (2009). Protein kinase G modulates human myocardial passive stiffness by phosphorylation of the titin springs. Circ Res.

[112] Kolijn D, Pabel S, Tian Y, Lodi M, Herwig M, Carrizzo A (2021). Empagliflozin improves endothelial and cardiomyocyte function in human heart failure with preserved ejection fraction via reduced pro-inflammatory-oxidative pathways and protein kinase Gα oxidation. Cardiovasc Res.

[113] Pabel S, Wagner S, Bollenberg H, Bengel P, Kovacs A, Schach C (2018). Empagliflozin directly improves diastolic function in human heart failure. Eur J Heart Fail.

[114] Rehman K, Akash MSH (2017). Mechanism of generation of oxidative stress and pathophysiology of type 2 diabetes mellitus: how are they interlinked?. J Cell Biochem.

[115] Stadler K (2012). Oxidative stress in diabetes. Adv Exp Med Biol.

[116] Luc K, Schramm-Luc A, Guzik TJ, Mikolajczyk TP (2019). Oxidative stress and inflammatory markers in prediabetes and diabetes. J Physiol Pharmacol.

[117] Daiber A, Xia N, Steven S, Oelze M, Hanf A, Kroller-Schon S (2019). New therapeutic implications of endothelial nitric oxide synthase (eNOS) function/dysfunction in cardiovascular disease. Int J Mol Sci.

[118] Karbach S, Wenzel P, Waisman A, Munzel T, Daiber A (2014). eNOS uncoupling in cardiovascular diseases–the role of oxidative stress and inflammation. Curr Pharm Des.

[119] Hasan R, Lasker S, Hasan A, Zerin F, Zamila M, Chowdhury FI (2020). Canagliflozin attenuates isoprenaline-induced cardiac oxidative stress by stimulating multiple antioxidant and anti-inflammatory signaling pathways. Sci Rep.

[120] Hasan R, Lasker S, Hasan A, Zerin F, Zamila M, Parvez F (2020). Canagliflozin ameliorates renal oxidative stress and inflammation by stimulating AMPK-Akt-eNOS pathway in the isoprenaline-induced oxidative stress model. Sci Rep.

[121] Sun X, Han F, Lu Q, Li X, Ren D, Zhang J (2020). Empagliflozin ameliorates obesity-related cardiac dysfunction by regulating sestrin2-mediated AMPK-mTOR signaling and redox homeostasis in high-fat diet-induced obese mice. Diabetes.

[122] Anavi S, Tirosh O (2020). iNOS as a metabolic enzyme under stress conditions. Free Radic Biol Med.

[123] Liu MM, Shi J (2020). Physiological and pathological effects of nitric oxide and nitric oxide synthase in oral cavity. Zhonghua Kou Qiang Yi Xue Za Zhi.

[124] Berka V, Liu W, Wu G, Tsai AL (2014). Comparison of oxygen-induced radical intermediates in iNOS oxygenase domain with those from nNOS and eNOS. J Inorg Biochem.

[125] Wu F, Tyml K, Wilson JX (2008). iNOS expression requires NADPH oxidase-dependent redox signaling in microvascular endothelial cells. J Cell Physiol.

[126] Chocry M, Leloup L (2020). The NADPH oxidase family and its inhibitors. Antioxid Redox Signal.

[127] Liu X, Zhu Q, Zhang M, Yin T, Xu R, Xiao W (2018). Isoliquiritigenin ameliorates acute pancreatitis in mice via inhibition of oxidative stress and modulation of the Nrf2/HO-1 pathway. Oxid Med Cell Longev.

[128] Liu X, Lin X, Zhang S, Guo C, Li J, Mi Y (2018). Lycopene ameliorates oxidative stress in the aging chicken ovary via activation of Nrf2/HO-1 pathway. Aging.

[129] Hu L, Tian K, Zhang T, Fan CH, Zhou P, Zeng D (2019). Cyanate induces oxidative stress injury and abnormal lipid metabolism in liver through Nrf2/HO-1. Molecules.

[130] Kitada M, Ogura Y, Monno I, Koya D (2019). Sirtuins and type 2 diabetes: role in inflammation, oxidative stress, and mitochondrial function. Front Endocrinol.

[131] Zhang B, Zhai M, Li B, Liu Z, Li K, Jiang L (2018). Honokiol ameliorates myocardial ischemia/reperfusion injury in type 1 diabetic rats by reducing oxidative stress and apoptosis through activating the SIRT1-Nrf2 signaling pathway. Oxid Med Cell Longev.

[132] Ren BC, Zhang YF, Liu SS, Cheng XJ, Yang X, Cui XG (2020). Curcumin alleviates oxidative stress and inhibits apoptosis in diabetic cardiomyopathy via Sirt1-Foxo1 and PI3K-Akt signalling pathways. J Cell Mol Med.

[133] Kim JW, Lee YJ, You YH, Moon MK, Yoon KH, Ahn YB (2019). Effect of sodium-glucose cotransporter 2 inhibitor, empagliflozin, and α-glucosidase inhibitor, voglibose, on hepatic steatosis in an animal model of type 2 diabetes. J Cell Biochem.

[134] Mohamed HE, Asker ME, Keshawy MM, Hasan RA, Mahmoud YK (2020). Inhibition of tumor necrosis factor-α enhanced the antifibrotic effect of empagliflozin in an animal model with renal insulin resistance. Mol Cell Biochem.

[135] Klotz LO, Sánchez-Ramos C, Prieto-Arroyo I, Urbánek P, Steinbrenner H, Monsalve M (2015). Redox regulation of FoxO transcription factors. Redox Biol.

[136] Ljubkovic M, Gressette M, Bulat C, Cavar M, Bakovic D, Fabijanic D (2019). Disturbed fatty acid oxidation, endoplasmic reticulum stress, and apoptosis in left ventricle of patients with type 2 diabetes. Diabetes.

[137] Yu ZW, Zhang J, Li X, Wang Y, Fu YH, Gao XY (2020). A new research hot spot: the role of NLRP3 inflammasome activation, a key step in pyroptosis, in diabetes and diabetic complications. Life Sci.

[138] Van Opdenbosch N, Lamkanfi M (2019). Caspases in cell death, inflammation, and disease. Immunity.

[139] Trang NN, Chung CC, Lee TW, Cheng WL, Kao YH, Huang SY (2021). Empagliflozin and liraglutide differentially modulate cardiac metabolism in diabetic cardiomyopathy in rats. Int J Mol Sci.

[140] Peña-Blanco A, García-Sáez AJ (2018). Bax, Bak and beyond—mitochondrial performance in apoptosis. Febs j.

[141] Kiraz Y, Adan A, Yandim MK, Baran Y (2016). Major apoptotic mechanisms and genes involved in apoptosis. Tumour Biol.

[142] Wang QL, Zhao L, Feng N, Zhou P, Wu Q, Fan R (2015). Lacidipine attenuates TNF-α-induced cardiomyocyte apoptosis. Cytokine.

[143] Yang CC, Chen YT, Wallace CG, Chen KH, Cheng BC, Sung PH (2019). Early administration of empagliflozin preserved heart function in cardiorenal syndrome in rat. Biomed Pharmacother.

[144] Lahnwong S, Palee S, Apaijai N, Sriwichaiin S, Kerdphoo S, Jaiwongkam T (2020). Acute dapagliflozin administration exerts cardioprotective effects in rats with cardiac ischemia/reperfusion injury. Cardiovasc Diabetol.

[145] Zhang J, Huang L, Shi X, Yang L, Hua F, Ma J (2020). Metformin protects against myocardial ischemia-reperfusion injury and cell pyroptosis via AMPK/NLRP3 inflammasome pathway. Aging.

[146] Hu R, Wang MQ, Ni SH, Wang M, Liu LY, You HY (2020). Salidroside ameliorates endothelial inflammation and oxidative stress by regulating the AMPK/NF-kappaB/NLRP3 signaling pathway in AGEs-induced HUVECs. Eur J Pharmacol.

[147] Ma C, Wang X, Xu T, Yu X, Zhang S, Liu S (2019). Qingkailing injection ameliorates cerebral ischemia-reperfusion injury and modulates the AMPK/NLRP3 inflammasome signalling pathway. BMC Complement Altern Med.

[148] Yue RC, Lu SZ, Luo Y, Wang T, Liang H, Zeng J (2019). Calpain silencing alleviates myocardial ischemia-reperfusion injury through the NLRP3/ASC/Caspase-1 axis in mice. Life Sci.

[149] Shao BZ, Xu ZQ, Han BZ, Su DF, Liu C (2015). NLRP3 inflammasome and its inhibitors: a review. Front Pharmacol.

[150] Shi J, Gao W, Shao F (2017). Pyroptosis: gasdermin-mediated programmed necrotic cell death. Trends Biochem Sci.

[151] Ji N, Qi Z, Wang Y, Yang X, Yan Z, Li M (2021). Pyroptosis: a new regulating mechanism in cardiovascular disease. J Inflamm Res.

[152] Ye Y, Bajaj M, Yang HC, Perez-Polo JR, Birnbaum Y (2017). SGLT-2 inhibition with dapagliflozin reduces the activation of the Nlrp3/ASC inflammasome and attenuates the development of diabetic cardiomyopathy in mice with type 2 diabetes. further augmentation of the effects with saxagliptin, a DPP4 inhibitor. Cardiovasc Drugs Ther.

[153] Chen H, Tran D, Yang HC, Nylander S, Birnbaum Y, Ye Y (2020). Dapagliflozin and ticagrelor have additive effects on the attenuation of the activation of the NLRP3 inflammasome and the progression of diabetic cardiomyopathy: an AMPK-mTOR interplay. Cardiovasc Drugs Ther.

[154] Levine B, Klionsky DJ (2004). Development by self-digestion: molecular mechanisms and biological functions of autophagy. Dev Cell.

[155] Mizushima N, Komatsu M (2011). Autophagy: renovation of cells and tissues. Cell.

[156] Delbridge LMD, Mellor KM, Taylor DJ, Gottlieb RA (2017). Myocardial stress and autophagy: mechanisms and potential therapies. Nat Rev Cardiol.

[157] Sciarretta S, Maejima Y, Zablocki D, Sadoshima J (2018). The role of autophagy in the heart. Annu Rev Physiol.

[158] Li Y, Yang P, Zhao L, Chen Y, Zhang X, Zeng S (2019). CD36 plays a negative role in the regulation of lipophagy in hepatocytes through an AMPK-dependent pathway. J Lipid Res.

[159] Gwinn DM, Shackelford DB, Egan DF, Mihaylova MM, Mery A, Vasquez DS (2008). AMPK phosphorylation of raptor mediates a metabolic checkpoint. Mol Cell.

[160] Arab HH, Al-Shorbagy MY, Saad MA (2021). Activation of autophagy and suppression of apoptosis by dapagliflozin attenuates experimental inflammatory bowel disease in rats: targeting AMPK/mTOR, HMGB1/RAGE and Nrf2/HO-1 pathways. Chem Biol Interact.

[161] Kim J, Kundu M, Viollet B, Guan KL (2011). AMPK and mTOR regulate autophagy through direct phosphorylation of Ulk1. Nat Cell Biol.

[162] Jiang K, Xu Y, Wang D, Chen F, Tu Z, Qian J (2022). Cardioprotective mechanism of SGLT2 inhibitor against myocardial infarction is through reduction of autosis. Protein Cell.

[163] Baartscheer A, Schumacher CA, Wust RC, Fiolet JW, Stienen GJ, Coronel R (2017). Empagliflozin decreases myocardial cytoplasmic Na(+) through inhibition of the cardiac Na(+)/H(+) exchanger in rats and rabbits. Diabetologia.

[164] Deng R, Jiang K, Chen F, Miao Y, Lu Y, Su F (2022). Novel cardioprotective mechanism for empagliflozin in nondiabetic myocardial infarction with acute hyperglycemia. Biomed Pharmacother.

[165] Lee WS, Yoo WH, Chae HJ (2015). ER stress and autophagy. Curr Mol Med.

[166] Ibrahim IM, Abdelmalek DH, Elfiky AA (2019). GRP78: a cell’s response to stress. Life Sci.

[167] Grootjans J, Kaser A, Kaufman RJ, Blumberg RS (2016). The unfolded protein response in immunity and inflammation. Nat Rev Immunol.

[168] Wu LD, Liu Y, Li F, Chen JY, Zhang J, Qian LL (2022). Glucose fluctuation promotes cardiomyocyte apoptosis by triggering endoplasmic reticulum (ER) stress signaling pathway in vivo and in vitro. Bioengineered.

[169] Hu H, Tian M, Ding C, Yu S (2018). The C/EBP homologous protein (CHOP) transcription factor functions in endoplasmic reticulum stress-induced apoptosis and microbial infection. Front Immunol.

[170] Kepp O, Semeraro M, Pedro JMBS, Bloy N, Buque A, Huang X (2015). eIF2α phosphorylation as a biomarker of immunogenic cell death. Semin Cancer Biol.

[171] Iurlaro R, Munoz-Pinedo C (2016). Cell death induced by endoplasmic reticulum stress. FEBS J.

[172] Ren FF, Xie ZY, Jiang YN, Guan X, Chen QY, Lai TF (2022). Dapagliflozin attenuates pressure overload-induced myocardial remodeling in mice via activating SIRT1 and inhibiting endoplasmic reticulum stress. Acta Pharmacol Sin.

[173] Wang CC, Li Y, Qian XQ, Zhao H, Wang D, Zuo GX (2022). Empagliflozin alleviates myocardial I/R injury and cardiomyocyte apoptosis via inhibiting ER stress-induced autophagy and the PERK/ATF4/Beclin1 pathway. J Drug Target.

[174] Chang WT, Lin YW, Ho CH, Chen ZC, Liu PY, Shih JY (2021). Dapagliflozin suppresses ER stress and protects doxorubicin-induced cardiotoxicity in breast cancer patients. Arch Toxicol.

[175] Hosokawa K, Takata T, Sugihara T, Matono T, Koda M, Kanda T (2019). Ipragliflozin ameliorates endoplasmic reticulum stress and apoptosis through preventing ectopic lipid deposition in renal tubules. Int J Mol Sci.

[176] Tang WHW, Li DY, Hazen SL (2019). Dietary metabolism, the gut microbiome, and heart failure. Nat Rev Cardiol.

[177] Hug H, Mohajeri MH, La Fata G (2018). Toll-like receptors: regulators of the immune response in the human gut. Nutrients.

[178] Cani PD, Amar J, Iglesias MA, Poggi M, Knauf C, Bastelica D (2007). Metabolic endotoxemia initiates obesity and insulin resistance. Diabetes.

[179] Jonsson AL, Bäckhed F (2017). Role of gut microbiota in atherosclerosis. Nat Rev Cardiol.

[180] Zhang Y, Wang Y, Ke B, Du J (2021). TMAO: how gut microbiota contributes to heart failure. Transl Res.

[181] Lee DM, Battson ML, Jarrell DK, Hou S, Ecton KE, Weir TL (2018). SGLT2 inhibition via dapagliflozin improves generalized vascular dysfunction and alters the gut microbiota in type 2 diabetic mice. Cardiovasc Diabetol.

[182] Shin NR, Lee JC, Lee HY, Kim MS, Whon TW, Lee MS (2014). An increase in the *Akkermansia* spp. population induced by metformin treatment improves glucose homeostasis in diet-induced obese mice. Gut..

[183] Li J, Lin S, Vanhoutte PM, Woo CW, Xu A (2016). *Akkermansia* muciniphila protects against atherosclerosis by preventing metabolic endotoxemia-induced inflammation in Apoe-/- mice. Circulation.

[184] Yang W, Yu T, Huang X, Bilotta AJ, Xu L, Lu Y (2020). Intestinal microbiota-derived short-chain fatty acids regulation of immune cell IL-22 production and gut immunity. Nat Commun.

[185] Ghosh SS, Wang J, Yannie PJ, Ghosh S (2020). Intestinal barrier dysfunction, LPS translocation, and disease development. J Endocr Soc.

[186] Hata S, Okamura T, Kobayashi A, Bamba R, Miyoshi T, Nakajima H (2022). Gut microbiota changes by an SGLT2 inhibitor, luseogliflozin, alters metabolites compared with those in a low carbohydrate diet in db/db mice. Nutrients.

[187] Deng X, Zhang C, Wang P, Wei W, Shi X, Wang P (2022). Cardiovascular benefits of empagliflozin are associated with gut microbiota and plasma metabolites in type 2 diabetes. J Clin Endocrinol Metab.

[188] Nozu T, Miyagishi S, Ishioh M, Takakusaki K, Okumura T (2021). Phlorizin attenuates visceral hypersensitivity and colonic hyperpermeability in a rat model of irritable bowel syndrome. Biomed Pharmacother.

[189] Ho HJ, Kikuchi K, Oikawa D, Watanabe S, Kanemitsu Y, Saigusa D (2021). SGLT-1-specific inhibition ameliorates renal failure and alters the gut microbial community in mice with adenine-induced renal failure. Physiol Rep.

[190] Mishima E, Fukuda S, Kanemitsu Y, Saigusa D, Mukawa C, Asaji K (2018). Canagliflozin reduces plasma uremic toxins and alters the intestinal microbiota composition in a chronic kidney disease mouse model. Am J Physiol Renal Physiol.

[191] Röder PV, Geillinger KE, Zietek TS, Thorens B, Koepsell H, Daniel H (2014). The role of SGLT1 and GLUT2 in intestinal glucose transport and sensing. PLoS ONE.

[192] Marfella R, D'Onofrio N, Trotta MC, Sardu C, Scisciola L, Amarelli C (2022). Sodium/glucose cotransporter 2 (SGLT2) inhibitors improve cardiac function by reducing JunD expression in human diabetic hearts. Metabolism.

[193] Verma S, Mazer CD, Yan AT, Mason T, Garg V, Teoh H (2019). Effect of empagliflozin on left ventricular mass in patients with type 2 diabetes mellitus and coronary artery disease: the EMPA-HEART cardiolink-6 randomized clinical trial. Circulation.

[194] Brown AJM, Gandy S, McCrimmon R, Houston JG, Struthers AD, Lang CC (2020). A randomized controlled trial of dapagliflozin on left ventricular hypertrophy in people with type two diabetes: the DAPA-LVH trial. Eur Heart J.

[195] Sposito AC, Breder I, Soares AAS, Kimura-Medorima ST, Munhoz DB, Cintra RMR (2021). Dapagliflozin effect on endothelial dysfunction in diabetic patients with atherosclerotic disease: a randomized active-controlled trial. Cardiovasc Diabetol.

[196] Leccisotti L, Cinti F, Sorice GP, D'Amario D, Lorusso M, Guzzardi MA (2022). Dapagliflozin improves myocardial flow reserve in patients with type 2 diabetes: the DAPAHEART Trial: a preliminary report. Cardiovasc Diabetol.

[197] Ott C, Jumar A, Striepe K, Friedrich S, Karg MV, Bramlage P (2017). A randomised study of the impact of the SGLT2 inhibitor dapagliflozin on microvascular and macrovascular circulation. Cardiovasc Diabetol.

[198] Paolisso P, Bergamaschi L, Santulli G, Gallinoro E, Cesaro A, Gragnano F (2022). Infarct size, inflammatory burden, and admission hyperglycemia in diabetic patients with acute myocardial infarction treated with SGLT2-inhibitors: a multicenter international registry. Cardiovasc Diabetol.

[199] Ridderstrale M, Andersen KR, Zeller C, Kim G, Woerle HJ, Broedl UC (2014). Comparison of empagliflozin and glimepiride as add-on to metformin in patients with type 2 diabetes: a 104-week randomised, active-controlled, double-blind, phase 3 trial. Lancet Diabetes Endocrinol.

[200] McDowell K, Welsh P, Docherty KF, Morrow DA, Jhund PS, de Boer RA (2022). Dapagliflozin reduces uric acid concentration, an independent predictor of adverse outcomes in DAPA-HF. Eur J Heart Fail.

[201] Davies MJ, Trujillo A, Vijapurkar U, Damaraju CV, Meininger G (2015). Effect of canagliflozin on serum uric acid in patients with type 2 diabetes mellitus. Diabetes Obes Metab.

[202] van Bommel EJM, Herrema H, Davids M, Kramer MHH, Nieuwdorp M, van Raalte DH (2020). Effects of 12-week treatment with dapagliflozin and gliclazide on faecal microbiome: Results of a double-blind randomized trial in patients with type 2 diabetes. Diabetes Metab.

[203] Li WJ, Chen XQ, Xu LL, Li YQ, Luo BH (2020). SGLT2 inhibitors and atrial fibrillation in type 2 diabetes: a systematic review with meta-analysis of 16 randomized controlled trials. Cardiovasc Diabetol.

[204] Salah HM, Al'Aref SJ, Khan MS, Al-Hawwas M, Vallurupalli S, Mehta JL (2022). Efficacy and safety of sodium-glucose cotransporter 2 inhibitors initiation in patients with acute heart failure, with and without type 2 diabetes: a systematic review and meta-analysis. Cardiovasc Diabetol.

[205] Yu YW, Zhao XM, Wang YH, Zhou Q, Huang Y, Zhai M (2021). Effect of sodium-glucose cotransporter 2 inhibitors on cardiac structure and function in type 2 diabetes mellitus patients with or without chronic heart failure: a meta-analysis. Cardiovasc Diabetol.

[206] Wilding JP, Rajeev SP, DeFronzo RA (2016). Positioning SGLT2 inhibitors/incretin-based therapies in the treatment algorithm. Diabetes Care.

[207] Georgianos PI, Agarwal R (2019). Ambulatory blood pressure reduction with SGLT-2 inhibitors: dose-response meta-analysis and comparative evaluation with low-dose hydrochlorothiazide. Diabetes Care.

[208] Dave CV, Schneeweiss S, Patorno E (2019). Comparative risk of genital infections associated with sodium-glucose co-transporter-2 inhibitors. Diabetes Obes Metab.

[209] Neal B, Perkovic V, Mahaffey KW, de Zeeuw D, Fulcher G, Erondu N (2017). Canagliflozin and cardiovascular and renal events in type 2 diabetes. N Engl J Med.

[210] Peters AL, Buschur EO, Buse JB, Cohan P, Diner JC, Hirsch IB (2015). Euglycemic diabetic ketoacidosis: a potential complication of treatment with sodium-glucose cotransporter 2 inhibition. Diabetes Care.

[211] Bailey CJ, Day C, Bellary S (2022). Renal protection with SGLT2 inhibitors: effects in acute and chronic kidney disease. Curr Diab Rep.

[212] Hahn K, Ejaz AA, Kanbay M, Lanaspa MA, Johnson RJ (2016). Acute kidney injury from SGLT2 inhibitors: potential mechanisms. Nat Rev Nephrol.

[213] Rivosecchi RM, Kellum JA, Dasta JF, Armahizer MJ, Bolesta S, Buckley MS (2016). Drug class combination-associated acute kidney injury. Ann Pharmacother.

[214] Wiviott SD, Raz I, Bonaca MP, Mosenzon O, Kato ET, Cahn A (2019). Dapagliflozin and cardiovascular outcomes in type 2 diabetes. N Engl J Med.

[215] Jackson K, Moseley KF (2020). Diabetes and bone fragility: SGLT2 inhibitor use in the context of renal and cardiovascular benefits. Curr Osteoporos Rep.

[216] Zannad F, Ferreira JP, Butler J, Filippatos G, Januzzi JL, Sumin M (2022). Effect of empagliflozin on circulating proteomics in heart failure: mechanistic insights into the EMPEROR programme. Eur Heart J.

[217] O'Meara E, McDonald M, Chan M, Ducharme A, Ezekowitz JA, Giannetti N (2020). CCS/CHFS heart failure guidelines: clinical trial update on functional mitral regurgitation, SGLT2 inhibitors, ARNI in HFpEF, and tafamidis in amyloidosis. Can J Cardiol.

[218] McDonald M, Virani S, Chan M, Ducharme A, Ezekowitz JA, Giannetti N (2021). CCS/CHFS heart failure guidelines update: defining a new pharmacologic standard of care for heart failure with reduced ejection fraction. Can J Cardiol.

